# Selective and Potent Peptide Binders of RNF43 for Wnt Signaling Inhibition

**DOI:** 10.1021/acscentsci.5c00744

**Published:** 2025-07-29

**Authors:** Sunhee Hwang, Paula Flórez Salcedo, Antonion Korcari, John M. Nicoludis, Estefania Martinez Valdivia, Lingling Peng, Aaron T. Balana, Justin Mak, Christopher M. Crittenden, Amin Famili, Peter Liu, David Castillo-Azofeifa, Rami N. Hannoush, Stephen E. Miller, Christina I. Schroeder, Xinxin Gao

**Affiliations:** Departments of †Peptide Therapeutics, ‡Regenerative Medicine, §Structural Biology, ∥Small Molecule Analytical Chemistry and Quality Control, ⊥Microchemistry, Proteomics and Lipidomics, Genentech Inc., South San Francisco, California 94080, United States

## Abstract

The Wnt/β-catenin pathway is critical in human tumor progression. Cell-surface transmembrane E3 ubiquitin ligase ring finger 43 negatively regulates Wnt signaling through ubiquitination of Wnt coreceptor Frizzled. Aberrant Wnt signaling through inactivating mutations of RNF43 has been identified in various forms of cancers, highlighting its significance in tumor biology. However, the precise mechanism underlying the function of RNF43 remains elusive, largely due to the absence of selective molecular tools allowing for detection or manipulation of endogenous RNF43. Here we present a series of disulfide-constrained peptides, including GUR-1.6.12.2, which exhibit high affinity and specificity against RNF43. GUR-1.6.12.2 can be used as a valuable research tool to delineate RNF43 activity in various contexts. We showcased its application in immunofluorescence, where RNF43 was detected in intestinal crypts using biotinylated GUR-1.6.12.2. We then combined experimental and computational structural approaches to propose a model of GUR-1.6.12.2 and its binding to RNF43. Importantly, we generated a functional RNF43-DCP by producing a hexavalent GUR-1.6.12.2 molecule, which exhibited inhibitory activity against Wnt signaling in cells by competing with R-spondin, a RNF43 ligand that potentiates signaling. The RNF43 binders presented here offer new opportunities for the research and development of anticancer therapies targeting Wnt signaling with improved selectivity.

## Graphical abstract

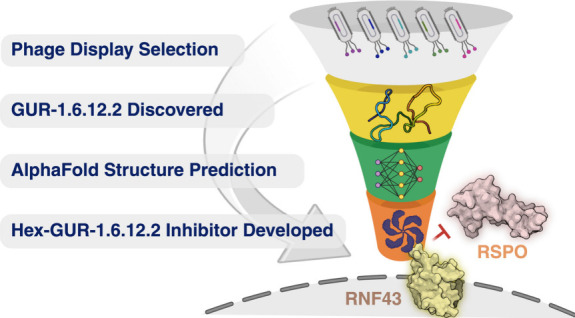

## Introduction

Discovered over four decades ago, Wnt signaling has been the focus of extensive research due to its essential role in human physiology and pathology.^1^ The Wnt/β-catenin pathway is integral to embryonic development as well as adult tissue homeostasis. Dysregulation of Wnt/β-catenin signaling can lead to an imbalance of normal cellular physiology and is implicated in a variety of diseases including cancer.^2,3^ Consequently, pharmacological modulation of Wnt signaling has emerged as an attractive approach for therapeutic applications, including tissue regeneration^4^ and cancer treatment.^5^ Wnt signaling is initiated at the plasma membrane, where a ternary complex is formed between Wnt ligands and their cell surface receptors, Frizzled and low-density lipoprotein receptor-related protein 5/6 (LRP5/6). Upon ligand binding, the Frizzled-LRP5/6 complex triggers the disassembly of the β-catenin destruction complex in cells, leading to cytosolic accumulation and subsequent nuclear translocation of β-catenin. Nuclear β-catenin acts as a transcriptional coactivator to regulate gene transcription essential for tissue self-renewal and cell proliferation.^6^ Abnormal activation of Wnt signaling is associated with early oncogenic events, highlighting the importance of negative regulation of the Wnt-β-catenin pathway in tumor suppression.

The membrane-bound E3 ubiquitin ligases, ring finger 43 (RNF43) and its homologue zinc and ring finger 3 (ZNRF3), are critical negative regulators of Wnt signaling.^7,8^ They downregulate Wnt signaling through ubiquitination of Frizzled, leading to its degradation. This inhibition can be counteracted by R-spondins (RSPO), which bind to leucine-rich repeat-containing G protein-coupled receptors 4–6 (LGR4–6) and RNF43/ZNRF3. This interaction initiates clearance of these E3 ligases from the membrane, ultimately enhancing Wnt signaling. RNF43 contains a protease-associated domain, a transmembrane region, and a cytoplasmic really interesting new gene (RING) domain that is responsible for its E3 ligase function.^9^ The pivotal role of the RNF43 in cancer has been established across various forms of cancers.^10^ Inactivating mutations of RNF43 have been identified in colorectal cancer, pancreatic ductal adenocarcinoma and endometrial cancer,^11,12^ underscoring its significance in oncogenesis. A deeper understanding of RNF43’s function in Wnt signaling suppression would offer valuable insights into the development of anticancer therapeutics targeting dysregulated Wnt signaling. Despite its recognized role in tumorigenesis, the precise mechanisms of action and biological function of RNF43 remain elusive. Additionally, the differential roles between ZNRF3 and RNF43 in Wnt signaling inhibition are not yet fully elucidated. This lack of clarity can be attributed, at least in part, to the limited availability of specific molecular tools capable of detecting or modulating endogenous ZNRF3 and RNF43 in their native cellular environment.^13,14^ The absence of such tools has left many critical questions regarding RNF43’s full scope of biological roles unanswered, highlighting this a crucial area for future development.

Over the past decade, cystine-knot peptides, a subgroup of disulfide- constrained peptides (DCPs), have garnered increased attention as templates for development of both research tools and potential peptide therapeutics.^15–17^ Cystine-knot peptides typically consist of 30–40 amino acid residues, including six cysteine residues with an intramolecular disulfide bonds arranged in a knotted conformation brought about by the well-known Cys I-Cys IV, Cys II-Cys V, Cys III-Cys VI disulfide connectivity. Many naturally occurring cystine-knot peptides have been isolated from diverse sources such as plants, fungi, bacteria and venom, and exhibit a broad spectrum of pharmacological activities.^18,19^ The high sequence diversity presented in their loop regions flanking the conserved cysteines suggests that novel biological functions can be engineered through sequence modification. Leveraging this unique feature, we previously developed an efficient and robust DCP phage library platform for identification of potent binders against various protein targets.^20–25^ The DCP phage libraries were constructed based on several DCP scaffolds, namely Ecballium elaterium trypsin inhibitor-II (EETI-II), AVR9, circulin-A, conotoxin-MVIIA, Huwentoxin, charybdotoxin, cellulose binding domain (CBD), Momordica charantia 1 (Mch1), gurmarin, Asteropsin-A, antimicrobial peptide-1 (AMP-1), potato carboxypeptidase inhibitor (CPI) and λ-MK1a.^20,23,24^ Twelve of these 13 wild-type DCPs, except CBD which contains only four cysteines, adopt a knotted conformation through conserved cysteines and are thus classified as cystine-knot peptides. By randomizing the hypervariable loops (individually or in combination) and displaying them on the M13 phage, we built a highly diverse library collection with varied shapes and topologies.

Using this platform, we have developed both functional and nonfunctional DCP binders against ZNRF3, derived from the libraries based on the scaffold isolated from the Scorpion (*Mesobuthus eupeus*) venom, λ-MK1a.^24^ The functional ZNRF3-DCPs promote ZNRF3 ubiquitination and cell surface clearance, resulting in Wnt signaling potentiation. The nonfunctional binders, on the other hand, serve as useful tools to study the roles of ZNRF3 in Wnt signaling.^24,26^ One caveat for these ZNRF3-DCPs is their sometimes unfavorably fast off-rates, potentially due to their smaller size relative to antibodies, which typically demonstrate longer residence time on their target. To address this, we explored the application of multivalent DCPs to enhance their residence time on ZNRF3. Through this approach, we generated a panel of multivalent ZNRF3-DCPs using a nonfunctional monomeric ZNRF3-DCP. The multivalent ZNRF3-DCPs demonstrated reduced off-rates when binding to ZNRF3 and were able to induce expansive growth of human intestinal organoids by promoting ZNRF3 membrane clearance. Notably, these DCPs demonstrated high specificity for ZNRF3, with no cross-reactivity toward its paralogue RNF43, presenting a novel strategy for dissecting the functional differences between the two E3 ligases.^24,26^ Despite these advancements, there remains a pressing need to develop tool molecules that can be used to investigate the functions of RNF43.

To further expand the molecular toolbox, here we report a series of selective DCP-based binders that specifically target RNF43, without cross-reactivity to ZNRF3. The lead DCP, GUR-1.6.12.2, binds to RNF43 with single-digit nanomolar affinity. Biotinylated GUR-1.6.12.2 was used in immunofluorescence assays to identify RNF43 localization in mouse intestines ex vivo. To characterize GUR-1.6.12.2 and elucidate its mechanism of action, we employed mass spectrometry, nuclear magnetic resonance (NMR) spectroscopy and computational modeling to elucidate the interaction between GUR-1.6.12.2 and RNF43. Furthermore, to enhance its functional activity, we re-engineered the monomeric DCP into a multivalent RNF43-DCP. This hexameric DCP enhanced RNF43 function by blocking its interaction with RSPO, effectively inhibiting Wnt signaling in cells. The molecular toolset described in this study, alongside previously developed ZNRF3-DCPs,^24^ enables in-depth investigation of RNF43/ZNRF3 regulation of Wnt signaling in their endogenous context.

## Results and Discussion

### Identification and Affinity Maturation of DCPs Targeting Human RNF43

To identify RNF43 binders, we initially performed primary panning using our DCP phage libraries, displayed on p8, against the extracellular domain (ECD) of the human RNF43 protein. After four rounds of selection, one potential hit, GUR-1, was identified from the libraries based on the gurmarin scaffold (Figure 1A). Gurmarin, a cystine-knot peptide with four loops and free N- and C- termini, was originally isolated from the leaves of the Asclepiad vine *Gymnema sylvestre*, an Indian-originated tree.^27,28^ Gurmarin selectively inhibits the neural response to sweet taste in rats but has no apparent effect in humans.^27–29^ The initial hit from primary phage-panning, GUR-1, contained sequence variations, as well an extended loop length in loop 4 compared to wild-type gurmarin (Figure 1B). Next, we chemically synthesized and folded GUR-1 using a previously established method.^20^ The synthetic peptide, containing three disulfide bonds, was purified using high-performance liquid chromatography (HPLC), and its purity was assessed by liquid chromatography–mass spectrometry (LC-MS). Finally, the binding affinity to RNF43 was determined using surface plasmon resonance (SPR), with a K_D_ value of approximately 1 μM (Figure 1C).

**Figure 1:**
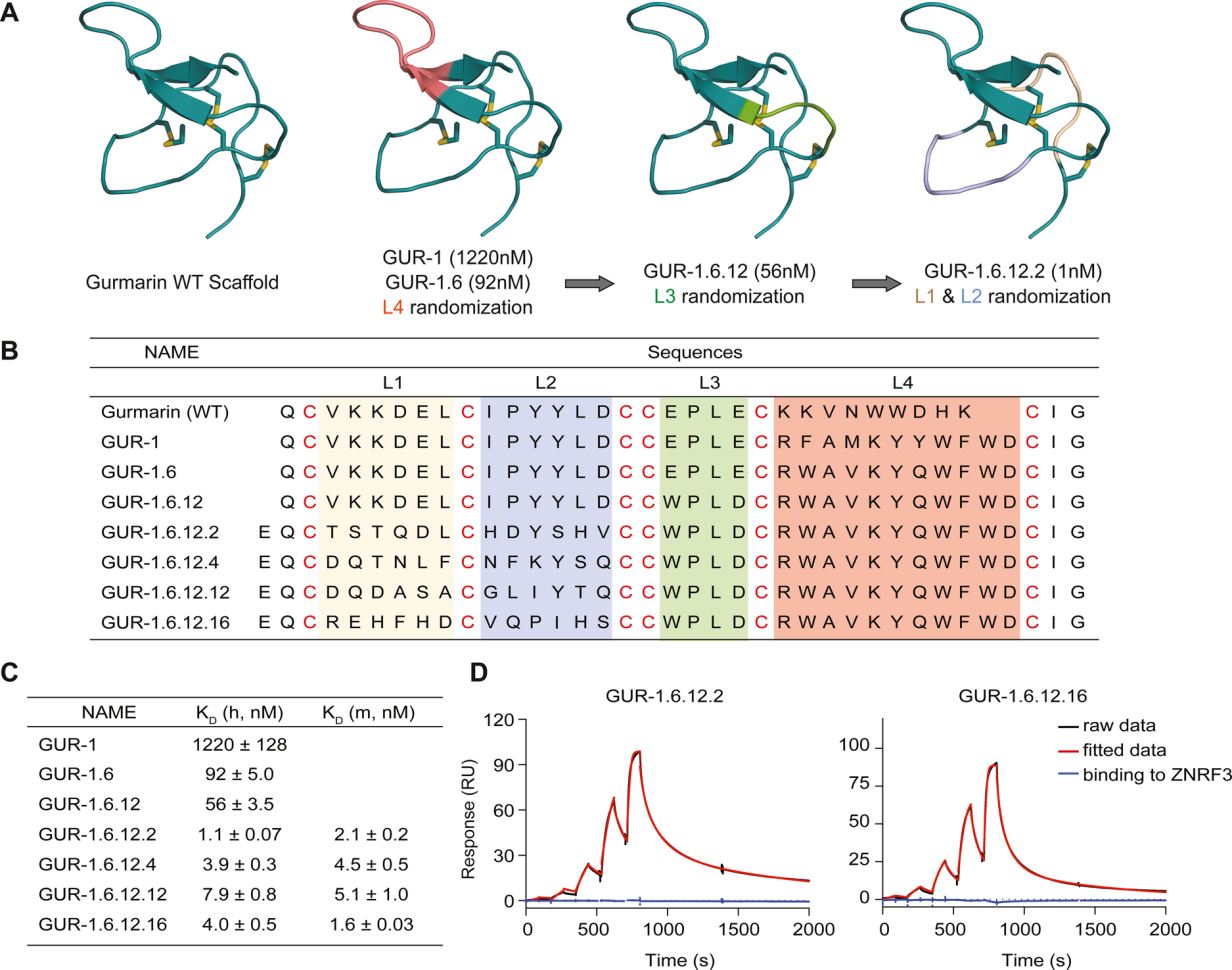
**Identification and affinity maturation of DCPs against human RNF43.** (A) Selection against RNF43. Three DCP phage libraries were generated based on gurmarin (PDB: 1C4E), with loop 4 (7, 9, or 11 amino acid length) randomized. The libraries were selected against human RNF43 to identify potential binders. (B) Affinity maturation to identify nanomolar binders against RNF43. Sequences and affinity of synthetic DCPs are shown in (B) and (C). The lead DCP molecules showed similar affinity against human and mouse RNF43 (C). h: human; m: mouse. (D) Representative SPR sensorgrams showing binding of the fourth-generation DCPs (GUR-1.6.12.2 and GUR-1.6.12.16) to human RNF43 immobilized on the sensor (black: raw data; red: fitted data; blue: ZNRF3 binding data). SPR was independently performed at least three times.

Given the modest binding affinity of GUR-1, we undertook affinity maturation to improve its interaction with RNF43. For the first round of affinity maturation, we performed soft randomization (see Methods for details regarding library construction) on the same loop, loop 4, displayed on the bacteriophage major protein p8 (Figure 1C). This round of affinity maturation resulted in a DCP molecule (GUR-1.6) with a 10-fold higher affinity for RNF43. GUR-1.6 differed in three residues in loop 4 compared to the sequence of GUR-1 (Figure 1B and C). This improvement in affinity enabled display of the DCP on the bacteriophage minor protein p3, where displayed DCPs act as monovalent binders, due to their low display levels compared to p8.^30^ The following hard randomization of the neighboring loop3 slightly improved the affinity of GUR-1.6 from the second generation, with only two amino acid residue altered (GUR-1.6.12) (Figure 1B). For the last round of affinity maturation, we focused on loops 1 and 2 simultaneously and constructed a phage library where 12 residues across loops 1 and 2 were subjected to hard randomization. This library, exhibiting much higher diversity than previous affinity maturation libraries, dramatically improved the affinity of the lead DCP by more than 50-fold, achieving single-digit nanomolar (nM) level (GUR-1.6.12.2, Figure 1A-D). GUR-1.6.12.2 also exhibited greater sequence variation, displaying differences in 11 residues. For the fourth generation of DCPs, we added an extra amino acid (glutamate, E) to the N-terminus of the peptides during chemical synthesis to enhance the production (synthesis and folding) yield by preventing the formation of pyroglutamate from the N-terminal Gln residue. Glutamate was selected to maintain consistency with the peptide sequences on the phage, as all peptide sequences start with “LE” on the p3 phage protein. Altogether, through three rounds of affinity maturation, we implemented changes in the amino acid composition across all four loops of GUR-1, resulting in more than 1000-fold improvement in its affinity against RNF43.

Fourth-generation GUR-1.6.12.2 and three of its analogues, GUR-1.6.12.4, GUR-1.6.12.12 and GUR-1.6.12.16, exhibited cross-species binding to both human and mouse RNF43 with similar affinity (Figure 1C). Importantly, they also demonstrated binding specificity for ligase RNF43, as indicated by their lack of binding to the structurally and functionally related Wnt signaling E3 ligase ZNRF3 (Figure 1D). These results demonstrate rapid development of selective high-affinity DCP binders against RNF43.

### Visualization of RNF43 in Mouse Intestine Using Biotinylated GUR-1.6.12.2

We hypothesized that GUR-1.6.12.2 could be a useful tool for a range of applications due to its high binding affinity and specificity. Therefore, as proof of concept we evaluated its utility in binding and immunofluorescence assays. We engineered an N-terminal biotinylated version of GUR-1.6.12.2 (bio-GUR-1.6.12.2) allowing for binding to streptavidin or neutravidin. We immobilized bio-GUR-1.6.12.2 on a SPR sensor coated with streptavidin and evaluated its affinity against RNF43. This method differs from our usual SPR protocols utilizing immobilized protein and untagged DCP. Bio-GUR-1.6.12.2 maintained the specificity (without binding to ZNRF3) observed for the original molecule GUR-1.6.12.2, albeit with decreased affinity (Figure 2A). This reduced affinity is likely due to the SPR sensor surface spatially hindering RNF43’s access to the binding sites of bio-GUR-1.6.12.2. Indeed, when we immobilized Fc-fused RNF43 on a protein A sensor, bio-GUR-1.6.12.2 maintained its high affinity (K_D_ = 0.5 ± 0.04 nM, Figure S1). We speculate that bio-GUR-1.6.12.2 with long PEG linkers^24,26^ would provide better access to the protein, therefore improving its binding affinity in assays where the peptide is immobilized on a surface. Nevertheless, the data confirms that bio-GUR-1.6.12.2 maintains high affinity and specificity against its target, making it suitable for assays probing the interaction of RNF43 and its binding partners.

**Figure 2:**
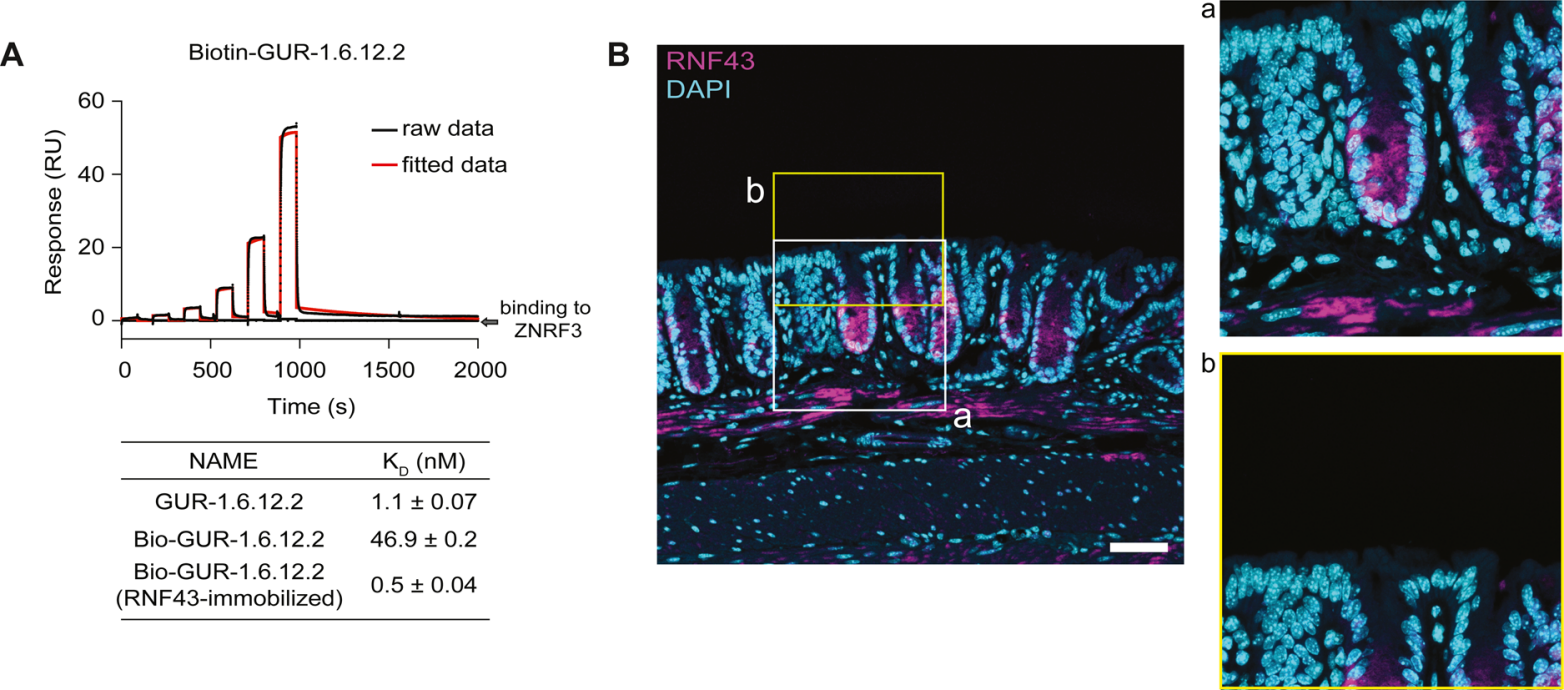
**Visualization of RNF43 in mouse intestine with biotinylated GUR-1.6.12.2.** (A) Representative SPR sensorgrams showing binding of N-terminal biotinylated GUR-1.6.12.2 (bio-GUR-1.6.12.2, immobilized onto the sensor) to human RNF43 (black: raw data; red: fitted data; blue: binding to ZNRF3). Bio-GUR-1.6.12.2 showed great specificity and nanomolar affinity against RNF43. SPR was independently performed at least three times. (B) Immunofluorescence staining of RNF43 using bio-GUR-1.6.12.2 in mouse colon. Scale bar: 40 μm.

We subsequently asked whether bio-GUR-1.6.12.2 could be used as a novel tool to visualize RNF43 in mouse colon tissues via immunofluorescence. Proper colon homeostasis and regeneration depends on Wnt signaling, and Wnt dysregulation in this tissue can lead to inflammatory disease or cancer, highlighting the applicability of the colon for visualizing key molecular components of Wnt signaling like RNF43. Previous studies have shown that Wnt signaling is active in the stem cell niche at the bottoms of intestinal crypt^31^ and that mRNA expression of RNF43 is localized to the lower regions of intestinal crypts.^7,32–34^ Using bio-GUR-1.6.12.2, we were able to detect RNF43 expression in mouse colon tissue. The staining showed high specificity to the bottom half of the intestinal crypts (Figure 2B), consistent with the RNF43 expression profile previously reported using mRNA-based approaches, i.e., *in situ* hybridization and RNA sequencing.^7,32–34^ Our findings demonstrate that bio-GUR-1.6.12.2 serves as an effective immunofluorescence reagent for probing RNF43. This innovative approach offers unparalleled insights into spatial expression patterns and allows for precise quantitative measurements of RNF43 within tissue samples. Additionally, this versatile tool holds promise for broader applications in protein interaction studies, including SPR, pull-down assay and enzyme-linked immunosorbent assay (ELISA).

### Characterization of GUR-1.6.12.2 and Its Interaction with RNF43

The successful use of GUR-1.6.12.2 as an imaging tool to visualize and investigate RNF43 encouraged us to further characterize this peptide and its interaction with the ligase. First, we determined the cysteine connectivity of GUR-1.6.12.2. GUR-1.6.12.2 only shares 51.3% homology with its parent molecule gurmarin, and it was therefore highly likely that GUR-1.6.12.2 did not possess the same knotted disulfide connectivity as the parent peptide following the use of directed evolution during discovery. Using an approach combining a two-step reduction and high-resolution mass spectrometry,^35^ we obtained a disulfide connectivity for GUR-1.6.12.2 comprising Cys I-Cys II, Cys III-Cys VI, Cys IV-Cys V (Figure 3A and Figure S2), which is different from gurmarin (Cys I-Cys IV, Cys II-Cys V, Cys III-Cys VI).

**Figure 3:**
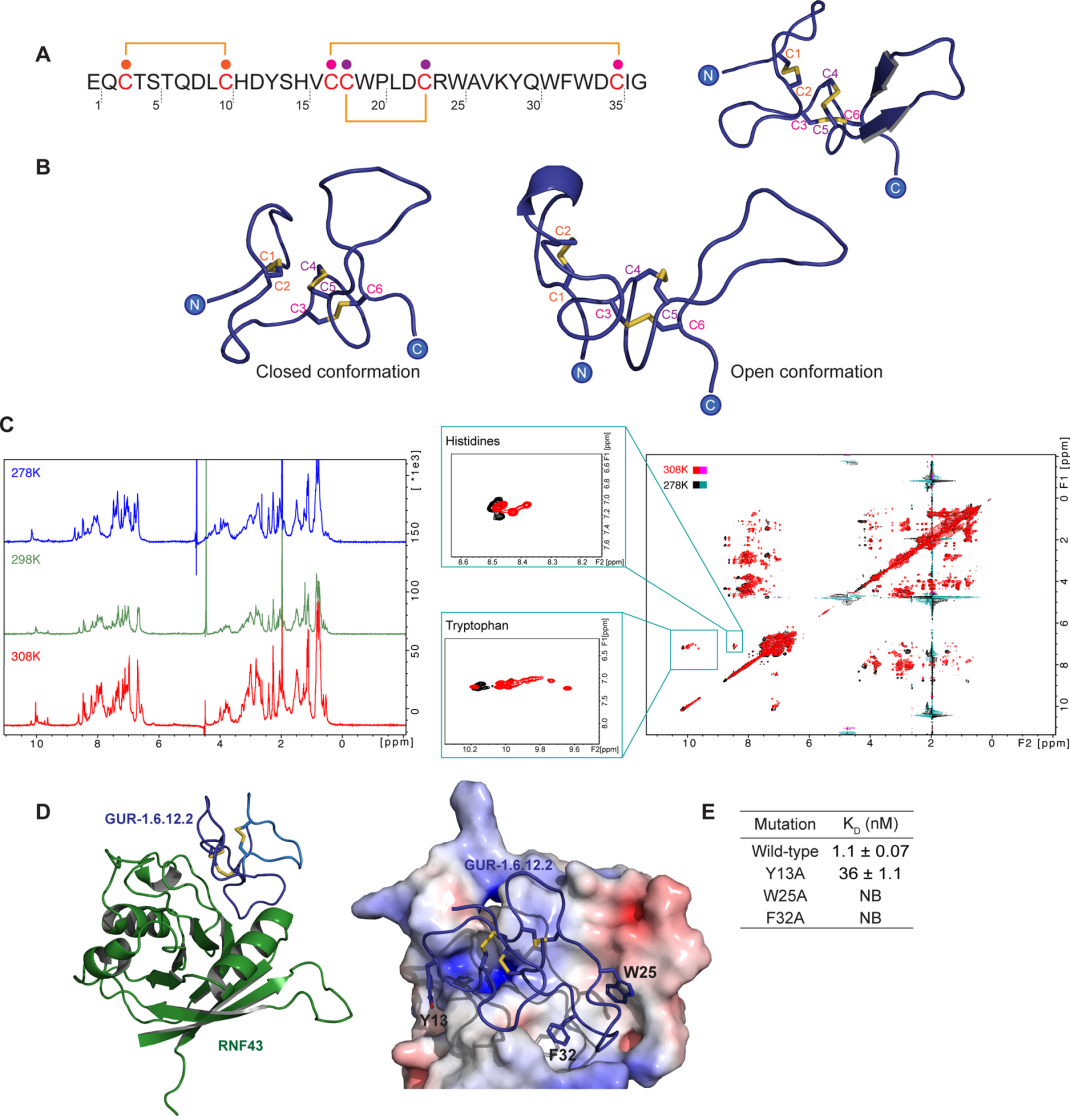
**Characterization of GUR-1.6.12.2.** (A) Experimentally determined disulfide connectivity of GUR-1.6.12.2 (Cys I–Cys II, Cys III–Cys VI, Cys IV–Cys V) and the KnotFold model of the peptide illustrating this connectivity. (B) Models of the two conformations derived from molecular dynamics simulation data: a closed conformation with a distance of less than 1 nm between Cys II and Cys IV, and an open conformation where the distance between the cysteines exceeds 1 nm. (C) NMR spectra collected to visualize the dynamic features of GUR-1.6.12.2 (1 mg/mL). 1D NMR spectra at three different temperatures, highlighting an increase in overlapping peaks with rising temperatures, and 2D DIPSI NMR spectra of GUR-1.6.12.2 at two different temperatures, focusing on the indole regions where multiple peaks were observed (green boxes). (D) The AlphaFold multimer model, incorporating KnotFold as a template, predicts the binding of GUR-1.6.12.2 to RNF43. Residues E1 to C10 are shown in light blue, and residues from H11 to G37 are shown in dark blue (the binding assay of these fragment peptides is provided in Figure S4). The model highlights key residues involved in the interaction with the receptor (the electrostatic surface of the RNF43 receptor was represented on the right). (E) Mutagenesis studies confirming the complex model. The key residues were mutated to alanine and the binding of the mutant peptides was determined using SPR. SPR was independently performed at least three times. NB: no binding.

To gain insight into the binding mode of GUR-1.6.12.2 to RNF43, we tried to cocrystallize RNF43 with GUR-1.6.12.2. However, despite multiple attempts using several conditions, we were unable to obtain any crystal structures with high resolution. We then turned to computational protein structure prediction tools to obtain structural information. However, using AlphaFold2, a structure of GUR-1.6.12.2 was predicted to contain native disulfide connectivity (Cys I-Cys IV, Cys II-Cys V, Cys III-Cys VI), most likely due to the presence of natural gurmarin-like peptide sequences in the multiple sequence alignment input to AlphaFold2.^36^ Interestingly, the top two AlphaFold2 models had drastically different topologies (Figure S3A and S3B). One model possesses some loop regions and a short antiparallel beta-strand, while the other model holds a series of short alpha-helices.

Given the incorrect connectivity of the AlphaFold2 predictions, we used KnotFold,^36^ a prediction tool that enables adding experimental disulfide connectivity data into computational structural predictions, to predict the structure of GUR-1.6.12.2. In order to determine which AlphaFold model to use as input in KnotFold we used circular dichroism (CD) spectroscopy to investigate the secondary structure of synthetic GUR-1.6.12.2 peptide and observed a signature consistent with beta-strand secondary structure including a negative peak at 218 nm (Figure S3C). In addition, no α-helical signature peaks at 208 or 222 nm were observed. This led us to use the features from AlphaFold2 model 1 (comprising beta-sheets) as the input model for KnotFold because of the consistency between the secondary structure characteristics of this model and the results from the CD experiment with synthetic peptide. Using the Cys I-Cys II, Cys III-Cys VI, Cys IV-Cys V connectivity as input in KnotFold, the program predicted a structure of GUR-1.6.12.2 containing beta-sheet secondary structural features correlating with the predicted AlphaFold model 1 as predicted (Figure 3A, Figure S3D and S3E). Upon inspecting the KnotFold model, it became clear that the connectivity established experimentally for GUR-1.6.12.2 disrupts the macrocycle brought about by Cys III-Cys VI connectivity found in knotted peptides. This intramolecular bond is crucial for the rigid structure of knottins.^37^ The nonknotted Cys I-Cys II, Cys III-Cys VI, Cys IV-Cys V connectivity can theoretically give rise a two-domain peptide with Cys I-Cys II comprising one domain and Cys III-Cys VI and Cys IV-Cys V comprising another. This led us to hypothesize that GUR-1.6.12.2 was flexible and able to adopt multiple conformations, which was further explored with Gaussian-accelerated Molecular Dynamics (GaMD) simulations.^38^ To analyze the dynamics of the peptide, we used MDTraj to calculate the distance between the Cα protons of Cys II and Cys V across all trajectories. The distance between these two cysteines ranged from 5.5 to 15.5 Å (Figure 3B and Figure S3F) suggesting two distinct conformations: a closed conformation, where the distance between the cysteines is less than 10 Å, and an open conformation, where the distance exceeds 10 Å (Figure 3B).

To validate the dynamic nature of GUR-1.6.12.2, we performed 1D and 2D NMR experiments. First, we performed 1D experiments of GUR-1.6.12.2 in deuterated acetonitrile and water at three different temperatures. The 1D spectra showed poor peak dispersion with multiple overlapping peaks (Figure 3C), a common set of features of highly dynamic and unfolded proteins. The increase in peak overlap with elevated temperature suggests that GUR-1.6.12.2 was more dynamic as energy, in the form of increased temperature, was introduced into the system. Similar results were observed in the 2D experiments, where the spectra show at least two different conformations based on the number of Trp and His ring protons observed, highlighted by green boxes in Figure 3C. Although GUR-1.6.12.2 only contains four tryptophan residues, more than four peaks were identified when examining the indoles in the NMR spectra. In addition, GUR-1.6.12.2 contains only two His residues, but at least four HE1 protons were observed. This supports the molecular dynamics observations, suggesting that GUR-1.6.12.2 is dynamic and can adopt multiple conformations in solution resolvable on a NMR time scale.

Despite the dynamic nature of the peptide, GUR-1.6.12.2 still exhibited high binding affinity to RNF43. To better understand the molecular determinants of this interaction, we used AlphaFold2-multimer.^36^ Since AlphaFold2 predicted the incorrect connectivity for GUR-1.6.12.2, we used the GUR-1.6.12.2 KnotFold model (Figure 3A), derived with input from experimental disulfide connectivity constraints, as a template for AlphaFold2 multimer predictions (Figure 3D).^39^ The model suggested that three residues (Y13, W25 and F32) on GUR-1.6.12.2 made crucial interactions with RNF43. When Y13, W25 or F32, located in a cavity surrounded by negatively charged residues, were mutated to alanine, the binding affinity was significantly reduced (a ∼ 40X reduction for Y13A or no binding for W25A and F32A, Figure 3E and Figure S4). Consistently, peptides composed of the first 10 amino acids (shown in light blue in Figure 3D), or the first 16 amino acids (shown in cyan in Figure S4) of GUR-1.6.12.2, comprising only the N-terminal Cys I-Cys II, showed no binding to RNF43. Another truncated peptide (shown in dark blue in Figure 3D), consisting of residues H11 to G37 of GUR-1.6.12.2, the C-terminal domain including Cys III-Cys VI and Cys IV-Cys V, was identified as the main driving force for binding to RNF43, with binding affinity similar to GUR-1.6.12.2 (Figure S4). The binding model was further supported by another SPR experiment showing that the binding affinity of GUR-1.6.12.2 remained unchanged against deglycosylated RNF43 (Figure S5A), as the predicted DCP binding site was distant from the two glycosylation sites (N62 and N92). In addition, we used a cross-linking mass spectrometry to experimentally validate the binding model. In this experiment, the cross-linkage sites between RNF43 and GUR-1.6.12.2 were identified with a high MeroX confidence score (Figure S5B). The N-terminus of GUR-1.6.12.2 cross-linked with K54, K181 and Y197 of RNF43. Given the spacer arm length of the BS3 cross-linker, these residues are considered to be in close proximity to the DCP binding site, which is consistent with the prediction proposed by the complex model.

Taken together, we combined biophysical, biochemical and computational approaches to reveal the dynamics of GUR-1.6.12.2 and its interaction mode with RNF43. This workflow offers an alternative strategy to study the mode of action of DCPs combining both computational and experimental methods to predict the binding site, when X-ray structures of DCP-protein complexes are unavailable.

### Hexameric GUR-1.6.12.2 Inhibits Wnt Signaling by Competing with RSPO

Developing an inhibitor of Wnt signaling activity through RNF43 would help understand the mechanistic role of RNF43 in cancer biology. The peptide-RNF43 binding model indicates that GUR-1.6.12.2 partially competes with RSPO for binding to RNF43 (Figure 4A). However, this partial competition was shown insufficient to induce Wnt signaling inhibition in the Wnt reporter assay using HEK293 TopBrite (TB) luciferase reporter cells (Figure S6A). Based on previous experience, we hypothesized that generating DCP multimers could result in improved affinity and activity compared to monomeric GUR-1.6.12.2 through altered kinetics or steric hindrance.^22,24,25^ To generate multivalent RNF43-DCPs, we chemically synthesized N-terminal azide-modified GUR-1.6.12.2 and conjugated this monomeric DCP to propargyl PEG linkers using the copper-catalyzed azide–alkyne cycloaddition reaction.^24^ Using this workflow, we produced a dimer (di-GUR-1.6.12.2, with a bivalent PEG5 linker) and a hexamer (hex-GUR-1.6.12.2, with a six-armed PEG linker) of GUR-1.6.12.2.

**Figure 4:**
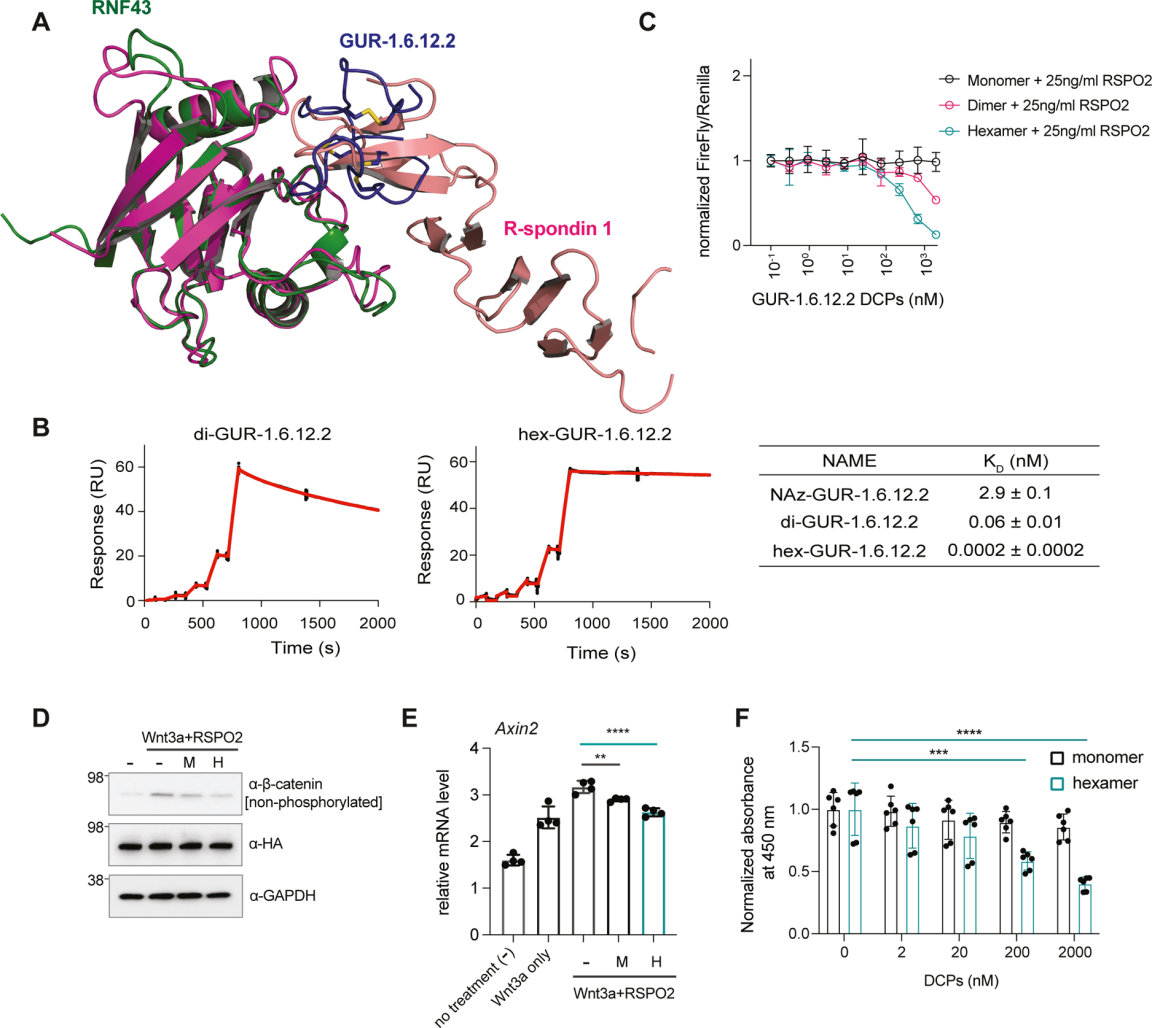
**Development of a bioactive RNF43-DCP molecule through multimerization.** (A) An overlay of the RNF43 (purple)-RSPO1 complex (PDB ID: 4KNG) with the AlphaFold prediction model (green) shows that the proposed binding site of GUR-1.6.12.2 (blue) partially overlaps with the RSPO1 epitope. Note the missing electron density for RSPO1 residues 133–138. (B) Representative SPR sensorgrams showing binding of the multivalent GUR-1.6.12.2 (di-GUR-1.6.12.2 and hex-GUR-1.6.12.2) to human RNF43 immobilized onto the sensor (black: raw data; red: fitted data). The binding affinity of GUR-1.6.12.2 was drastically improved (a 33-fold improvement for di-GUR-1.6.12.2 and a 10,000-fold improvement for hex-GUR-1.6.12.2). SPR was independently performed at least three times. NAz-1.6.12.2: the building block, GUR-1.6.12.2 with an N-terminal azide. (C) Multivalent GUR-1.6.12.2 molecules demonstrated functional activity in the Wnt report assay. The activity of di-GUR-1.6.12.2 and hex-GUR-1.6.12.2 was tested using the Wnt reporter assay. Data were normalized to negative controls. Presented are the mean ± SD from a representative experiment. The experiment was independently performed three times. (D) Western blot analysis showed a reduced level of active-β-catenin with hex-GUR-1.6.12.2 treatment. Representative data from three independent experiments are shown. (E) RT-qPCR analysis showed that hex-GUR-1.6.12.2 (10 nM) strongly inhibited the elevated *Axin2* mRNA level induced by RSPO2 in HEK293 cells overexpressing RNF43. GUR-1.6.12.2 (1 μM) demonstrated lower activity. Presented are the mean ± SD from four independent experiments. **: *p* < 0.005, ****: *p* < 0.0001. (F) Hex-GUR-1.6.12.2 competes with RSPO2 for binding to RNF43 in an ELISA where RNF43 was immobilized on the plate. Presented are mean ± SD from a representative experiment. The experiment was independently performed three times. ***: *p* < 0.0005, ****: *p* < 0.0001. One-way ANOVA with a multiple comparison test was used. The data were analyzed using Prism 10.

SPR experiments indicated that both molecules showed altered kinetics including substantially improved residence time and binding affinity to RNF43, with the hexamer demonstrating an apparent K_D_ value in the subpicomolar range, representing a 10,000-fold improvement over the monomer GUR-1.6.12.2 (Figure 4B). This is consistent with our previous studies where multivalency drastically improved the affinity of a ZNRF3-DCP toward the ligase.^24^ We then assessed the cellular activity of these multivalent GUR-1.6.12.2 DCPs. Di-GUR-1.6.12.2 and hex-GUR-1.6.12.2, but not the monomer GUR-1.6.12.2, were able to inhibit Wnt signaling in the reporter assay, in the presence of endogenous or exogenous RSPO2 (Figure 4C and Figure S6B). Cell viability was not affected, confirming both the specificity of the assay and nontoxicity of the multivalent GUR-1.6.12.2 (Figure S6C). We further confirmed the inhibitory effect of hex-GUR-1.6.12.2 using Western blot analysis where the level of β-catenin was analyzed with an antibody. In HEK293 cells transiently transfected with cDNA encoding full-length human RNF43, hex-GUR-1.6.12.2 caused a pronounced decrease in the level of active β-catenin in the presence of recombinant Wnt3a and RSPO2 (Figure 4D). Moreover, using RT-qPCR analysis, we demonstrated that stimulation with Wnt3a in HEK293 cells overexpressing RNF43 elevated the *Axin2* mRNA level, a marker for Wnt signaling activation. The addition of RSPO2 to the cells could further enhance this activation, presumably due to membrane clearance of RNF43. Treatment of these cells with hex-GUR-1.6.12.2 strongly reduced the elevated *Axin2* mRNA level induced by RSPO2, resulting in a ∼ 80% inhibition compared with cells treated with Wnt3a alone (Figure 4E). In contrast, the monomer GUR-1.6.12.2 exhibited lower activity. The more pronounced inhibitory effect of hex-GUR-1.6.12.2 was likely achieved through a stronger competition of hex-GUR-1.6.12.2 with RSPO, potentially brought about by avidity effect and steric hindrance, since we observed that hex-GUR-1.6.12.2 could disrupt the binding between RNF43 and RSPO2 potently in an ELISA experiment (Figure 4F).

Collectively, the data demonstrates that hex-GUR-1.6.12.2 has improved binding affinity and acts as enhanced negative regulators of Wnt signaling compared to the monomer. The antagonism displayed by hex-GUR-1.6.12.2 could be explained by the fact that the hexamer, with its increased size and higher affinity, disrupts the RNF43-RSPO2 interaction more potently than the monomer and rescues the function of RNF43 to inhibit Wnt signaling.

## Conclusion

Our powerful phage-display platform has previously demonstrated the ability of generating high affinity DCPs for a range of targets.^20–25^ In this study, using this DCP phage display platform,^20,23,24^ we developed monomeric gurmarin-derived DCPs with low nanomolar affinity for cell-surface transmembrane E3 ubiquitin ligase RNF43. We present here the development, structural characterization and application of the DCPs and their ability to detect and/or modulate the function of RNF43. The lead DCP, GUR-1.6.12.2, exhibited high affinity and more importantly, selectivity for RNF43 over ZNRF3, and a hexamer version of GUR-1.6.12.2 showed subpicomolar affinity and functional activity for RNF43. We also showed that monomeric GUR-1.6.12.2 can be used as a research tool to detect the expression of RNF43 in intestinal tissue.

We combined experimental data and computational predictions to investigate the structure of GUR-1.6.12.2 and to model the interaction of GUR-1.6.12.2 with RNF43. Experimentally derived disulfide connectivity information showed that GUR-1.6.12.2 lost the cysteine knot connectivity during affinity maturation. The disulfide connectivity of GUR-1.6.12.2 was incorporated in KnotFold to generate a model of GUR-1.6.12.2. Molecular dynamics demonstrated that the peptide was dynamic appearing to exist in an open and closed conformation. The dynamic nature of the peptide was subsequently confirmed by NMR. We then used the KnotFold model as a template in AlphaFold-Multimer to generate a binding model prediction of how the peptide interacts with RNF43 which was confirmed with mutational and cross-linking data. Our work demonstrates that the synergy between experimental data and computational structure prediction can be particularly useful when structural complexes are challenging to determine experimentally using traditional structural methods.

Our model suggested that GUR-1.6.12.2 interacts with RNF43 in a manner that could partially block RSPO binding. Although we observed weak inhibition of Wnt signaling by monomeric GUR-1.6.12.2 in several assays (Figure 4D-F), it was not potent enough to show functional effects in the TopBrite cellular assay. We then leveraged the avidity effect and steric bulk brought about by multimerization of individual DCPs to generate both dimeric and hexameric GUR-1.6.12.2 DCPs. Our hex-GUR-1.6.12.2 resulted in a functionally active DCP that was able to partially compete with RSPO in cells to inhibit Wnt signaling showcasing the power of the avidity effect and demonstrating multimerization as an effective strategy for generating functional DCPs as was previously seen for ZNRF3.^24^

Modulation of Wnt signaling is an attractive approach for future tumor treatment, both alone and in combination therapy. Inhibitors that enhance the activity of negative Wnt signaling regulators such as ZNRF3 and RNF43 have emerged as a novel strategy for developing specialized treatments. However, controlled and selective inhibition of the ligases has remained challenging.^13,14^ Even though it is well established that inactivating mutations of RNF43 is associated with oncogenesis, molecules that can inhibit RNF43 specifically have not been developed thus far. Recently developed ZNRF3/RNF43 modulators rely heavily on RSPO-derived binding domains and are therefore unable to discriminate between the two ligases.^14^ The panel of DCPs presented here, with their superb specificity and unique modularity, could facilitate research deciphering unknown functional differences between ZNRF3 and RNF43 in oncogenesis, thereby offering an alternative strategy to develop anticancer therapeutics targeting Wnt signaling ligases in distinct tissues with less adverse side effect.^40^

## Materials and Methods

### Protein Biotinylation

ECD of the human RNF43 protein was incubated with EZ-link-NHS-PEG4-biotin (Thermo Fisher, 1:1, 50 μM) for 1 h at room temperature (RT). The reaction was quenched by addition of 10 mM Glycine, followed by dialysis into phosphate buffered saline (PBS) overnight at 4 °C. Finally, the biotinylated protein was analyzed using LC-MS to ensure that each protein is labeled with 1–2 biotin molecules.^20,23,24^

### DCP Phage Library Sorting and Affinity Maturation

For primary phage panning, naïve DCP phage libraries were pooled into four groups according to the scaffolds.^20,23,24^ The libraries were subjected to four rounds of solution selection against biotinylated human RNF43 ECD (Genentech) as described previously.^20,23,24^ After the fourth round, phages displaying DCPs binding to the target were analyzed by phage spot ELISA followed by Sanger sequencing. Select DCPs were synthesized and oxidized as described previously.^20,23,24^ For hard randomization, each nucleotide position contained a mixture of randomized nucleotides. For soft randomization, each nucleotide position contained a mixture of 70% parent nucleotide sequence and 10% of each of the other nucleotides, yielding a library with approximately 50% parent amino acids at each position^41^ The libraries were constructed on gene 8 or gene 3 of the M13 bacteriophages.

### Phage Spot ELISA

To perform the assay, a 384 well plate was coated with 2 μg/mL of neutravidin (in PBS) overnight at 4 °C. The plate was then blocked with PBS containing 0.5% bovine serum albumin (BSA) and incubated with biotinylated protein or BSA for 20 min at RT. Individual phages displaying DCPs were added to the plate and incubated for 30 min at RT. The plate was then washed and incubated with a horseradish peroxidase (HRP)-conjugated anti-M13 antibody (CAB-655M, Creative Diagnostics) for 30 min at RT, followed by development and quenching with 1 M H_3_PO_4_. The plate was read at 450 nm to calculate the signal over noise ratios.^20,23,24^

### Peptide Synthesis

Linear peptides were synthesized on a peptide synthesizer (CS136M, CS Bio) using standard fluorenylmethoxycarbonyl (Fmoc) synthesis on 2-chlorotrityl resin. Peptides were cleaved from resin and purified by reverse phase HPLC. To form disulfide bonds, linear peptides were folded using either A) 0.1 M ammonium bicarbonate (NH_4_HCO_3_), pH 9.0, 2 mM reduced glutathione, 0.5 mM oxidized glutathione, 4% DMSO or B) 0.1 M NH_4_HCO_3_, pH 8.0, 1 mM reduced glutathione, 50% DMSO at 0.5 mg/mL for 24 h at RT with shaking. The folded peptides were purified with a C18 reversed phase (RP)-HPLC column and quality controlled by LC-MS. Peptide content was calculated by amino acid analysis, performed by CS Bio (Milpitas, CA).^20,23,24^

Oxidative folding of truncated GUR-1.6.12.2 E1-C10 and E1-V16 was achieved by mixing 1 equiv of dry purified linear peptide, oxidized and reduced glutathione (100 equiv, each) and dissolving to a concentration of 0.1 mg/mL of peptide with 100 mM NH_4_HCO_3_, at pH 8.5. The mixture was stirred at RT for 4 h. Folding of truncated peptide GUR-1.6.12.2 H11-G37 was achieved by mixing 1 equiv of purified linear peptide, oxidized and reduced glutathione (10 and 100 equiv, respectively) in 6 M guanidium chloride (GnHCl) at a concentration of 1 mg/mL of peptide. This mixture was added to a solution of 1 M GnHCl/0.4 M ammonium acetate (NH_4_OAc) at pH 8.0, for a final peptide concentration of 0.1 mg/mL and stirred at 4 °C for 4 days. The folding reactions were tracked using an Agilent 1290 LC-MS system. Once folding was complete, the reaction mixture was adjusted to pH 3 using trifluoroacetic acid (TFA). The single major product of each reaction was isolated by semipreparative RP-HPLC on a Teledyne instrument using a Phenomenex Luna C18, 250 × 10 mm column. A gradient of 0–30% acetonitrile/0.1% TFA/H_2_O was used for peptides E1-C10 and E1-V16, and 5–40% acetonitrile/0.1% TFA/H_2_O for peptide H11-G37. Successful disulfide bond formation was confirmed by LC-MS, and 1D and 2D ^1^H NMR.

### Synthesis of Multivalent DCPs

Dimeric and hexameric DCPs were synthesized as previously described.^24^ Stock solutions of 60 mM tris­(3-hydroxypropyltriazolylmethyl)­amine (THPTA), 20 mM copper­(II) sulfate (CuSO_4_), 100 mM aminoguanidine, 100 mM sodium ascorbate (all in water), and 5 mM bis-propargyl-PEG5 dimer linker (in a 50:50 mixture of dimethylformamide (DMF) and water) was prepared. 7.6 mg of N-terminal azide tagged DCP (Naz-GUR-1.6.12.2) was added to a flask and dissolved by adding 150 μL DMF and 667 μL water, followed by the addition of 75 μL 1 M ammonium bicarbonate buffer (pH 8.0). 150 μL of the PEG5 linker stock was added to the reaction flask. 150 μL CuSO_4_ and 150 μL THPTA stocks were premixed, transferred to the reaction flask, followed by the addition of 150 μL aminoguanidine stock. The reaction was initiated by the addition of 150 μL sodium ascorbate stock, and capped with a rubber stopper. Final concentrations for all the reagents were: 1 mM Naz-GUR-1.6.12.2; 0.5 mM dimer linker; 10 mM sodium ascorbate; 10% DMF; 2 mM CuSO_4_; 6 mM THPTA; 10 mM aminoguanidine, 50 mM ammonium bicarbonate pH 8.0; 1.5 mL total reaction volume. The reaction was monitored and verified by LC-MS to be complete after 1 h. Copper capture resin (Silicycle) was added to the reaction flask and diluted with 3.5 mL of 1% TFA in water, filtered with a 0.45 μm syringe filter, and was injected to a prep HPLC system (Agilent). Fractions with high purity (assessed by LC-MS) were combined, frozen, and lyophilized. Hexamer ligand was made through a similar protocol, using 1.5 mM Naz-GUR-1.6.12.2 and 0.2 mM hexamer linker, with all other reagents kept at the same concentration and volume. Both linkers were purchased from BroadPharm (dimer linker: bis-propargyl-PEG5, cat# BP-20660; hexamer linker: PEG3-bis­(Amino-Tri-(Propargyl-PEG2-ethoxymethyl)-methane), cat# BP-25649).

### Mapping of Disulfide Connectivity by Mass Spectrometry

GUR-1.6.12.2 (1 mg) was dissolved in 500 μL of 10 mM glycine buffer, pH 3.0, containing 20% (v/v) acetonitrile. Tris­(2-carboxyethyl)­phosphine (TCEP) (2 equiv) was added and incubated at 37 °C for 30 min. The formation of major [M+2] and [M+4] peaks were detected by analytical LC-MS (Agilent 6230 LC/TOF, Acquity UPLC BEH C18 column, 1.7 μm, 2.1 × 50 mm, 0.8 mL/min, 10 min gradient from 5 to 60% aqueous acetonitrile containing 0.05% TFA). *N*-ethylmaleimide (NEM) was then added to a final concentration of 50 mM, and the reaction was incubated at RT for 1 h to cap the free thiols. The crude material was purified by HPLC (C18 column, 5 μm, 10 × 250 mm, 100 Å, 4 mL/min, 20 min gradient from 25 to 45% aqueous acetonitrile containing 0.05% TFA), the [M+2 × NEM] and [M+4 × NEM] species were collected separately and lyophilized. The two samples were dissolved in 100 μL of 50% aqueous acetonitrile separately and fully reduced with 15 mM dithiothreitol (DTT) at 37 °C for 1 h. Iodoacetamide (IAA) was added to a final concentration of 40 mM to alkylate all remaining free thiols at pH 8.5, followed by incubation at RT for 1 h. The two generated samples [M+2 × NEM+4IAA] and [M+4 × NEM+2IAA] were neutralized to pH 6–7 with 2% acetic acid and analyzed by LC-MS. Separations were conducted using a Vanquish UHPLC system equipped with a Vanquish Polar Advantage II column (2.1 × 250 mm, 2.2 μm) heated to 50 °C (Thermo Fisher Scientific, USA). Mobile phase A was 0.1% formic acid in water and mobile phase B was 0.1%v/v formic acid in acetonitrile. The chromatographic separation was carried out at 0.5 mL/min going from 23 to 36% B over 18 min; total program time including regeneration and re-equilibration was 25 min. Mass analyses were conducted using an Orbitrap Fusion Lumos Tribrid Mass Spectrometer (Thermo Fisher Scientific, USA) with positive electrospray ionization. MS1 spectra were collected with quadrupole isolation (*m*/*z* 400–2000) with an AGC target of 8E4 ions and detected by the Orbitrap at 50K resolution. For MS2 fragmentation studies, precursor ions were isolated using 5 Th windows with a predicted AGC target of 2E6 ions and fragmented using Electron-Transfer Dissociation with default settings and supplemental Higher-Energy Collision Dissociation activation (EThcD) at 28, 30, 33, and 35 V. MS2 spectra were detected by the Orbitrap at 120 K resolution and 2 microscans.

Protein Metrics software (Boston, MA, USA) was used to analyze the data and identify derivatized cysteines. Spectra were manually reviewed, and isotope distributions were compared to theoretical distributions. To investigate the presence of alternate peptide sequence configurations that may be coeluting, alternate sequences were forced to be the correct configuration, and MS2 spectra were manually examined. Since terminal sequence coverage around each cysteine is required for accurate identification, alternate sequences without this coverage were eliminated. For identification settings, MS2 mass accuracy error limit was set to 20 ppm and the *m*/*z* signal-to-noise filter was set to greater than 3.

### SPR

SPR measurement was carried out using a Biacore S200 (Cytiva) instrument, with PBS-T (PBS with 0.05% Tween 20, pH 7.4) as the buffer. Biotinylated protein was captured on three sensor channels of Series S SA Sensors (Cytiva). The sensors were then blocked with 0.1 mg/mL biotin. DCPs were injected onto the sensor in a 3× concentration series. The data were double-referenced by subtracting the signal from a sensor channel coated with biotin only and the signal from a buffer injection. For data analysis, binding data were fitted using the manufacturer’s software.^20,23,24^

### Wnt Reporter Assay

The assay was performed as described previously.^24^ Briefly, HEK293 cells with firefly luciferase-based Wnt reporter (TOPbrite) and pRL-SV40 Renilla luciferase (Promega) were seeded to the 96-well plate (Corning, cat#3903) and incubated for 24 h. 50 ng/mL recombinant human Wnt3a protein (5036-WN, R&D systems) was then added to the plate, in the absence or presence of DCPs. Cells were then subjected to the Dual-Luciferase Reporter Assay kit (E2920, Promega) according to the manufacturer’s instructions after a 6 h incubation. Signals were measured on a PerkinElmer EnVision multilabel reader. The ratio of firefly over Renilla luminescence was calculated and normalized to controls.

### Immunofluorescence

Whole colons were isolated, rinsed with PBS to remove any potential fecal material, and fixed in 4% paraformaldehyde for 24 h at RT before being placed in 70% ethanol (EtOH) at RT and processed for paraffin embedding. Sections were cut at 6 μm thickness and dried in a 60 °C oven for 1 h. Next, sections were rehydrated in two xylene washes (5 min per wash at RT), followed by two 100% EtOH washes (10 min per wash at RT and 2X H_2_O washes (5 min per wash at RT). After rehydration, antigen retrieval was performed using tris-EDTA (1 mM at pH 9) in a pressure cooker for 30 min. Next, sections were blocked with 5% normal goat serum (NGS) (005–000–121, Jackson ImmunoResearch) for 1 h at RT. Then, sections were incubated with the DCP at 1 μM for 72 h at 4 °C. After 72 h, sections were washed with PBST (9809S, Cell Signaling) for three times (1 h per wash at RT). For the last wash, sections were left in PBST for 24 h at RT to further decrease background staining. After 24 h, sections were treated with 1% hydrogen peroxide (H_2_O_2_) for 15 min at RT, followed by 3X PBST washes (5 min per wash at RT). VECASTAIN Elite ABC kit (PK-6100, Vector Laboratories) was used on each section for 30 min at RT, and sections were washed with PBST twice (5 min per wash at RT). Slides were incubated with TSA Cyanine 3 Tyramide Reagent kit (1:100; SAT704B001EA, PerkinElmer) for 5 min at RT and washed with PBST twice (5 min per wash at RT). Finally, sections were counterstained with 4’,6-diamidino-2-phenylindole (DAPI) (1:10000; D9542, Sigma-Aldrich) in PBST for 5 min at RT, washed with PBST twice (5 min per wash at RT), and mounted using Prolong Gold Antifade (P36930, ThermoFisher Scientific). Fluorescence images of tissue sections were acquired with a Leica SP8 confocal microscope. Images were processed with the open-source platform Fiji.

### RT-qPCR

2.0 × 10^5^ HEK293 Topbrite luciferase reporter cells were seeded onto 12-well plates. The next day, the cells were treated with 50 ng/mL recombinant Wnt3a (5036-WN-010), 25 ng/mL recombinant RSPO2 (3266-RS-025, R&D systems), and monomeric or hexameric DCPs for 6 h. Cells were gently washed with PBS, and RNAs were isolated using the RNeasy kit (Qiagen), according to the manufacturer’s instructions. Briefly, cells were lysed in RLT buffer including β-mercaptonethanol, followed by addition of 70% ethanol. The sample was then transferred to the column provided in the kit. After several washing steps, RNA was eluted using RNase-free water. Real-time PCR reactions were performed with the Taqman RNA-to-C_T_ 1-step kit (Applied Biosystems) by preparing samples as follows (total 10 μL reaction). 5.0 μL of 2× TaqMan RT-PCR mix, 0.5 μL of 20× TaqMan gene expression probe, 0.25 μL of 40× TaqMan RT enzyme mix, 50 ng of RNA (2 μK of 25 ng/μL), and 2.25 μL of nuclease-free water. The reaction was initiated at 48 °C for reverse transcription for 15 min, followed by 45 amplification cycles (activation of AmpliTaq Gold DNA polymerase at 95 °C for 10 min, denaturation at 95 °C for 15 s, and anneal/extend at 95 °C for 1 min). The assay was run on QuanStudio 7 Flex Real-Time PCR systems (Thermo Fisher Scientific). Relative RNA levels were calculated using the ΔΔC_*T*_ method and normalized to the level of housekeeping gene human HPRT1 within the same sample and further normalized to the sample from cells with no treatment. The Taqman RNA-to C_T_ 1-step kit (4392653) and gene expression probes (HPRT1-FAM: Hs02800695_m1, Axin2-FAM: Hs01063168-m1) were purchased from Thermo Fisher Scientific.

### Western Blot Analysis

The cells were treated as described above. After 6 h of incubation, cells were gently washed with PBS and lysed in 100 μL of lysis buffer (PBS, 1% (v/v) Triton X-100 and protease and phosphatase inhibitor cocktail (Thermo Fisher Scientific)). The collected lysate was centrifuged for 5 min at 18,400 *g* to remove cell debris, and the supernatant was used to determine the total protein concentration via a BCA assay (Thermo Fisher Scientific). A total of 10 μg of protein was loaded onto an SDS-PAGE (4–12%, invitrogen), transferred to a nitrocellulose membrane, and probed with an anti-HA antibody (3724S, Cell Signaling) and an anti-nonphospho (active) β-catenin antibody (8814S, Cell Signaling) to assess full-length RNF43 expression and active β-catenin levels.

### Peptide Modeling

AlphaFold 2 version 2.3.1 was used to generate initial models. These models were used as input for KnotFold^36^ where disulfide connectivity was enforced based on experimental data, and models were generated using the BreakN MSA coevolution parameter. The KnotFold model was then used as a template to run AlphaFold multimer using ColabFold.

### NMR Spectroscopy

NMR spectra were collected on a Bruker 500 MHz spectrometer equipped with a TCI cryo probe 1.7. The peptide sample was prepared at a concentration of 1 mg/mL in a mixture of deuterated acetonitrile (CD_3_CN) and water (30:70). 1D and 2D ^1^H NMR experiments, including DIPSI sequences for homonuclear Hartman-Hahn transfer (70 ms mixing time) using an excitation sculpting water suppression protocol, were performed at 278, 298, and 308 K. The spectral width was 13.1544 ppm with 64 scans. The 1D ^1^H NMR experiments focused on identifying chemical shifts, while 2D DIPSI experiments employed a mixing time optimized for efficient magnetization transfer. Data were processed using TopSpin software, with spectral referencing to water.

### Deglycosylation

Protein deglycosylation was performed on a small scale using Deglycosylation Mix II (P6044, NEB), according to the manufacturer’s instructions. Briefly, the biotinylated RNF43 was incubated with Deglycosylation Mix II for 1 h at RT. Then the reaction mixture was transferred to 37 °C and further incubated overnight. Following incubation, the reaction mixture was analyzed by mobility shift on SDS-PAGE gels. After dialysis, the protein was then used for further applications.

### Cross-Linking Mass Spectrometry

RNF43 and GUR-1.6.12.2 were preincubated for 1 h on ice at a 1:10 ratio for complex formation. Then, a 50× excess amount of BS3 (bis­(sulfosuccinimidyl)­suberate) (21580, Thermo Fisher) was added to initiate cross-linking reaction. The mixture was incubated for 15 min at RT and quenched with 1 M Tris buffer (final concentration 50 μM). The mixture was then reduced with 10 mM dithiothreitol (DTT) for 1 h 37 °C and alkylated with 20 mM iodoacetamide (IAA) for another 30 min at RT. The mixture was then digested with trypsin (Promega), chymotrypsin (Roche) and elastase (Promega) in 25 mM ammonium bicarbonate, pH 8.0, overnight at 37 °C. The digestion was quenched with 2% formic acid, desalted using Pierce C18 spin tips, and dried in a speed-vac. The desalted peptides were suspended in 2% acetonitrile with 0.1% formic acid, and 1/50 of the digests were subjected to LC-MS/MS analysis on a Thermo Fisher Scientific Orbitrap-Fusion Lumos Tribrid mass spectrometer. The gradient was supplied using a Dionex U3000 nLC system with a C18 BEH column and consisted of 0–40% acetonitrile in 2% acetonitrile with 0.1% formic acid over 1 h at 0.4 μL/min. Data were acquired under data-dependent mode with 2-s duty cycle acquisition using OT-HCD/CID fragmentation. The parent ion resolution was 120,000 fwhm, and OT-CID/HCD fragmentation was performed at a resolution 15,000 fwhm. The raw data files were collected for data analysis. MeroX open-source software was used for data processing.

### CD Spectroscopy

CD experiments were performed to determine the secondary structure of GUR-1.6.12.2. A 5 μM peptide solution was prepared in a buffer containing 50 mM Bis-Tris Propane (BTP) and 150 mM sodium fluoride (NaF), pH 7.4. Wavelength spectra were measured from 180 to 260 nm at RT using a 0.1 mm path length quartz cell. The mean residue ellipticity [θ] was calculated based on the measured ellipticity, the length of the cell, the molar concentration, and the number of residues in a peptide. CD spectra were collected on Jasco *J*-815 CD spectrometer.

### Competition ELISA

A 384-well plate was coated with 2 μg/mL of neutravidin (in PBS) and incubated overnight at 4 °C. The plate was then blocked with PBS containing 0.5% BSA, and biotinylated RNF43 was immobilized onto the plate for 20 min at RT. A mixture of 1 nM His-tagged RSPO2 and 0–2 μM DCPs was added to the plate and incubated for 45 min at RT. The plate was then washed and incubated with an HRP-conjugated His antibody (ab1187, Abcam). The bound RSPO2 was detected using a peroxidase substrate and TMB peroxidase substrate mix (SeraCare), followed by quenching with 1 M H_3_PO_4_. The plate was read at 450 nm.

## Supplementary Material





## Notes

Supplementary Figures showing (1) SPR sensorgram of bio-GUR-1.6.12.2 binding to Fc-fused RNF43, (2) assessment of disulfide connectivity of GUR-1.6.12.2, (3) modeling of GUR-1.6.12.2 and predictions of its binding to RNF43, (4) representative SPR sensorgrams of GUR-1.6.12.2 mutant peptides, (5) supportive evidence for the complex model of GUR-1.6.12.2 with RNF43 and (6) activities of GUR-1.6.12.2 in cellular assays (PDF)
Source Data including LC-MS analysis for all the peptides used in the manuscript and original Western blot images (PDF)

## References

[1] Holzem M., Boutros M., Holstein T. W. (2024). The Origin
and Evolution of Wnt Signalling. Nat. Rev. Genet..

[2] Polakis P. (2012). Drugging Wnt
Signalling in Cancer: Wnt Signalling. EMBO J..

[3] Zhang Y., Wang X. (2020). Targeting the Wnt/β-Catenin Signaling Pathway in Cancer. J. Hematol. Oncol..

[4] Clevers H., Loh K. M., Nusse R. (2014). Stem Cell Signaling.
An Integral
Program for Tissue Renewal and Regeneration: Wnt Signaling and Stem
Cell Control. Sci. (N. York, NY).

[5] Anastas J. N., Moon R. T. (2013). WNT Signalling Pathways
as Therapeutic Targets in Cancer. Nat. Rev.
Cancer.

[6] Steinhart Z., Angers S. (2018). Wnt Signaling in Development and Tissue Homeostasis. Development.

[7] Koo B.-K., Spit M., Jordens I., Low T. Y., Stange D. E., van de Wetering M., van Es J. H., Mohammed S., Heck A. J. R., Maurice M. M., Clevers H. (2012). Tumour Suppressor RNF43 Is a Stem-Cell
E3 Ligase That Induces Endocytosis of Wnt Receptors. Nature.

[8] Hao H.-X., Xie Y., Zhang Y., Charlat O., Oster E., Avello M., Lei H., Mickanin C., Liu D., Ruffner H., Mao X., Ma Q., Zamponi R., Bouwmeester T., Finan P. M., Kirschner M. W., Porter J. A., Serluca F. C., Cong F. (2012). ZNRF3 Promotes Wnt
Receptor Turnover in an R-Spondin-Sensitive Manner. Nature.

[9] Chen P.-H., Chen X., Lin Z., Fang D., He X. (2013). The Structural
Basis of R-Spondin Recognition by LGR5 and RNF43. Genes Dev..

[10] Zhan T., Rindtorff N., Boutros M. (2017). Wnt Signaling in Cancer. Oncogene.

[11] Giannakis M., Hodis E., Mu X. J., Yamauchi M., Rosenbluh J., Cibulskis K., Saksena G., Lawrence M. S., Qian Z. R., Nishihara R., Van Allen E. M., Hahn W. C., Gabriel S. B., Lander E. S., Getz G., Ogino S., Fuchs C. S., Garraway L. A. (2014). RNF43 Is
Frequently Mutated in Colorectal and Endometrial
Cancers. Nat. Genet..

[12] Jiang X., Hao H.-X., Growney J. D., Woolfenden S., Bottiglio C., Ng N., Lu B., Hsieh M. H., Bagdasarian L., Meyer R., Smith T. R., Avello M., Charlat O., Xie Y., Porter J. A., Pan S., Liu J., McLaughlin M. E., Cong F. (2013). Inactivating Mutations
of RNF43 Confer Wnt Dependency in Pancreatic Ductal Adenocarcinoma. Proc. Natl. Acad. Sci. U. S. A..

[13] Tsukiyama T. (2024). New Insights
in Ubiquitin-Dependent Wnt Receptor Regulation in Tumorigenesis. Vitr. Cell. Dev. Biol. - Anim..

[14] Wolf L., Angers S. (2024). Get Your Receptors
in a Knot with New Wnt Signaling
Agonists. Cell Chem. Biol..

[15] Tammineni R., Gulati P., Kumar S., Mohanty A. (2020). An Overview of Acyclotides:
Past, Present and Future. Phytochemistry.

[16] Ho T. N. T., Turner A., Pham S. H., Nguyen H. T., Nguyen L. T. T., Nguyen L. T., Dang T. T. (2023). Cysteine-Rich
Peptides: From Bioactivity
to Bioinsecticide Applications. Toxicon.

[17] Tyler T. J., Durek T., Craik D. J. (2023). Native
and Engineered Cyclic Disulfide-Rich
Peptides as Drug Leads. Molecules.

[18] Daly N. L., Craik D. J. (2011). Bioactive Cystine
Knot Proteins. Curr. Opin. Chem. Biol..

[19] Craik D. J., Daly N. L., Waine C. (2001). The Cystine Knot Motif in Toxins
and Implications for Drug Design. Toxicon.

[20] Gao X., Kaluarachchi H., Zhang Y., Hwang S., Hannoush R. N. (2024). A Phage-Displayed
Disulfide Constrained Peptide Discovery Platform Yields Novel Human
Plasma Protein Binders. PLoS One.

[21] Li Y., Wei Y., Ultsch M., Li W., Tang W., Tombling B., Gao X., Dimitrova Y., Gampe C., Fuhrmann J., Zhang Y., Hannoush R. N., Kirchhofer D. (2024). Cystine-Knot Peptide Inhibitors of
HTRA1 Bind to a Cryptic Pocket within the Active Site Region. Nat. Commun..

[22] Hansen S., Zhang Y., Hwang S., Nabhan A., Li W., Fuhrmann J., Kschonsak Y., Zhou L., Nile A. H., Gao X., Piskol R., de Sousa e Melo F., de Sauvage F. J., Hannoush R. N. (2022). Directed Evolution Identifies High-Affinity Cystine-Knot
Peptide Agonists and Antagonists of Wnt/β-Catenin Signaling. Proc. Natl. Acad. Sci. United States Am..

[23] Zhou L., Cai F., Li Y., Gao X., Wei Y., Fedorova A., Kirchhofer D., Hannoush R. N., Zhang Y. (2024). Disulfide-Constrained
Peptide Scaffolds Enable a Robust Peptide-Therapeutic Discovery Platform. PLoS One.

[24] Kschonsak Y. T., Gao X., Miller S. E., Hwang S., Marei H., Wu P., Li Y., Ruiz K., Dorighi K., Holokai L., Perampalam P., Tsai W.-T. K., Kee Y.-S., Agard N. J., Harris S. F., Hannoush R. N., de Sousa e Melo F. de
S. e. (2024). Potent and Selective
Binders of the E3 Ubiquitin Ligase ZNRF3 Stimulate Wnt Signaling and
Intestinal Organoid Growth. Cell Chem. Biol..

[25] Thakur A. K., Miller S. E., Liau N. P. D., Hwang S., Hansen S., de Sousa e Melo F., Sudhamsu J., Hannoush R. N. (2023). Synthetic Multivalent
Disulfide-Constrained Peptide Agonists Potentiate Wnt1/β-Catenin
Signaling via LRP6 Coreceptor Clustering. ACS
Chem. Biol..

[26] Hwang S., Balana A. T., Martin B., Clarkson M., Di Lello P., Wu H., Li Y., Fuhrmann J., Dagdas Y., Holder P., Schroeder C. I., Miller S. E., Gao X. (2024). Bioproduction Platform
to Generate Functionalized Disulfide-Constrained Peptide Analogues. ACS Bio Med. Chem. Au.

[27] Imoto T., Miyasaka A., Ishima R., Akasaka K. (1991). A Novel Peptide Isolated
from the Leaves of Gymnema Sylvestre—I. Characterization and
Its Suppressive Effect on the Neural Responses to Sweet Taste Stimuli
in the Rat. Comp. Biochem. Physiol. Part A:
Physiol..

[28] Kamei K., Takano R., Miyasaka A., Imoto T., Hara S. (1992). Amino Acid
Sequence of Sweet-Taste-Suppressing Peptide (Gurmarin) from the Leaves
of Gymnema Sylvestre. J. Biochem..

[29] Fletcher J. I., Dingley A. J., Smith R., Connor M., Christie M. J., King G. F. (1999). High-resolution Solution Structure of Gurmarin, a Sweet-taste-suppressing
Plant Polypeptide. Eur. J. Biochem..

[30] Fuh G., Sidhu S. S. (2000). Efficient Phage Display of Polypeptides Fused to the
Carboxy-terminus of the M13 Gene-3 minor Coat Protein. FEBS Lett..

[31] Barker N., van Es J. H., Kuipers J., Kujala P., van den
Born M., Cozijnsen M., Haegebarth A., Korving J., Begthel H., Peters P. J., Clevers H. (2007). Identification of Stem Cells in Small
Intestine and Colon by Marker Gene Lgr5. Nature.

[32] Burclaff J., Bliton R. J., Breau K. A., Ok M. T., Gomez-Martinez I., Ranek J. S., Bhatt A. P., Purvis J. E., Woosley J. T., Magness S. T. (2022). A Proximal-to-Distal Survey of Healthy Adult Human
Small Intestine and Colon Epithelium by Single-Cell Transcriptomics. Cell. Mol. Gastroenterol. Hepatol..

[33] Xie L., Fletcher R. B., Bhatia D., Shah D., Phipps J., Deshmukh S., Zhang H., Ye J., Lee S., Le L., Newman M., Chen H., Sura A., Gupta S., Sanman L. E., Yang F., Meng W., Baribault H., Vanhove G. F., Yeh W.-C., Li Y., Lu C. (2022). Robust Colonic
Epithelial Regeneration and Amelioration of Colitis via FZD-Specific
Activation of Wnt Signaling. Cell. Mol. Gastroenterol.
Hepatol..

[34] Loregger A., Grandl M., Mejías-Luque R., Allgäuer M., Degenhart K., Haselmann V., Oikonomou C., Hatzis P., Janssen K.-P., Nitsche U., Gradl D., van den Broek O., Destree O., Ulm K., Neumaier M., Kalali B., Jung A., Varela I., Schmid R. M., Rad R., Busch D. H., Gerhard M. (2015). The E3 Ligase
RNF43 Inhibits Wnt
Signaling Downstream of Mutated β-Catenin by Sequestering TCF4
to the Nuclear Membrane. Sci. Signal..

[35] Chin S., Chen T., Hannoush R. N., Crittenden C. M. (2021). Tracking
Internal and External Ions for Constrained Peptides Leads to Enhanced
Sequence Coverage and Disulfide Bond Deciphering. J. Pharm. Biomed. Anal..

[36] Gerlach G. J., Nicoludis J. M. (2024). KnotFold: Improving Peptide Structure Predictions with
Simulated Coevolution. PRX Life.

[37] Kintzing J. R., Cochran J. R. (2016). Engineered Knottin
Peptides as Diagnostics, Therapeutics,
and Drug Delivery Vehicles. Curr. Opin. Chem.
Biol..

[38] Miao Y., Feher V. A., McCammon J. A. (2015). Gaussian Accelerated Molecular Dynamics:
Unconstrained Enhanced Sampling and Free Energy Calculation. J. Chem. Theory Comput..

[39] Mirdita M., Schütze K., Moriwaki Y., Heo L., Ovchinnikov S., Steinegger M. (2022). ColabFold: Making Protein Folding Accessible to All. Nat. Methods.

[40] Bugter J. M., Fenderico N., Maurice M. M. (2021). Mutations and Mechanisms
of WNT Pathway
Tumour Suppressors in Cancer. Nat. Rev. Cancer.

[41] Tonikian R., Zhang Y., Boone C., Sidhu S. S. (2007). Identifying Specificity
Profiles for Peptide Recognition Modules from Phage-Displayed Peptide
Libraries. Nat. Protoc..

