# Supplementary material for: Selective and Potent Peptide Binders of RNF43 for Wnt Signaling Inhibition
Source: ACS Cent Sci. 2025 Jul 29;11(9):1670–81. doi: 10.1021/acscentsci.5c00744 (PMC12464775; doi:10.1021/acscentsci.5c00744)
Supplement: Supplementary file 1 [file oc5c00744_si_001.pdf]

Supporting Information for Publication

Selective and Potent Peptide Binders of RNF43 for Wnt Signaling Inhibition

Sunhee Hwang[1], Paula Flórez Salcedo[1], Antonion Korcari[2], John M. Nicoludis[3], Estefania Martinez Valdivia[1], Lingling Peng[1], Aaron T. Balana[1], Justin Mak[4], Christopher M. Crittenden[4], Amin Famili[4], Peter Liu[5], David Castillo-Azofeifa[2], Rami N. Hannoush[1], Stephen E. Miller[1], Christina I. Schroeder[1], and Xinxin Gao[1*]

Departments of [1]Peptide Therapeutics, [2]Regenerative Medicine, [3]Structural Biology, [4]Small Molecule Analytical Chemistry and Quality Control, [5]Microchemistry, Proteomics and Lipidomics, Genentech Inc., South San Francisco, CA 94080, USA

*Correspondence: gao.xinxin@gene.com

Address: 1 DNA Way, South San Francisco, CA 94080, USA

Keywords: disulfide-constrained peptide, Wnt signaling, E3 ubiquitin ligase, RNF43

Table of Contents

Figure S1 (Page S3)

- SPR sensorgram of bio-GUR-1.6.12.2 binding to Fc-fused RNF43

Figure S2 (Page S4)

- Disulfide Connectivity of GUR-1.6.12.2 determined by Electron-Transfer/Higher-Energy Collision Dissociation

Figure S3 (Page S6)

- Modeling of GUR-1.6.12.2 and predictions of its binding to RNF43

Figure S4 (Page S8)

- Representative SPR sensorgrams of GUR-1.6.12.2 mutant peptides

Figure S5 (Page S10)

- Supportive evidence for the complex model of GUR-1.6.12.2 with RNF43

Figure S6 (Page S11)

- Activities of GUR-1.6.12.2 in cellular assays

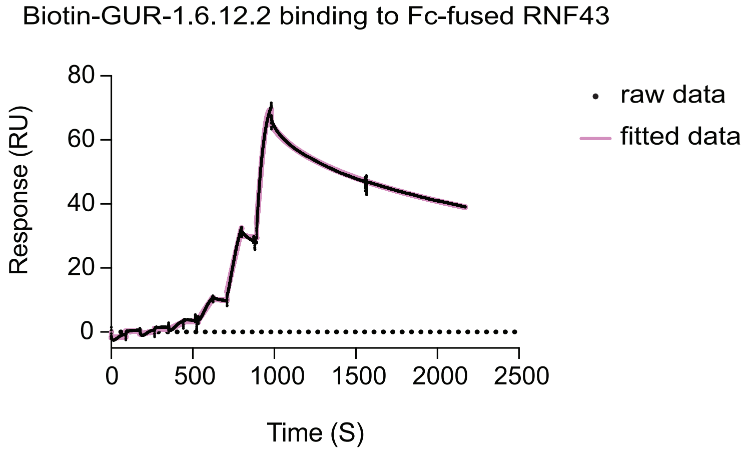

Figure S1. SPR sensorgram of bio-GUR-1.6.12.2 binding to Fc-fused RNF43 (immobilized on the sensor). SPR was independently performed at least three times (black: raw data; red: fitted data).

A GUR-1.6.12.2 - 2 NEM. Observed m/z 1251.5226, z=4 (0.45 ppm error).

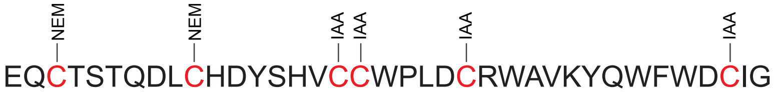

b3 identifes C3-NEM

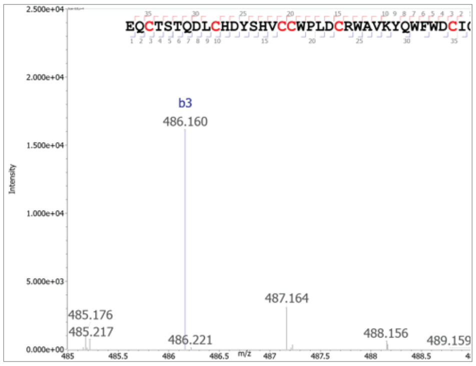

b10 identifes C10-NEM

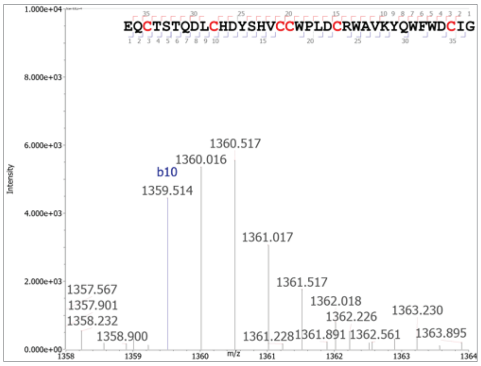

B GUR-1.6.12.2 - 4 NEM. Observed m/z 1285.5344, z=4 (-0.55 ppm error).

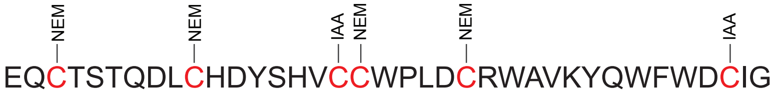

y3 identifies C35-IAA

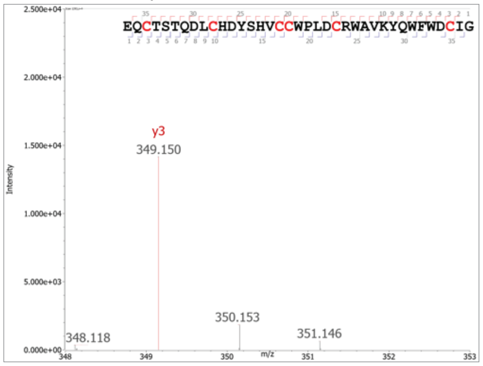

y20++ identifes C18-NEM

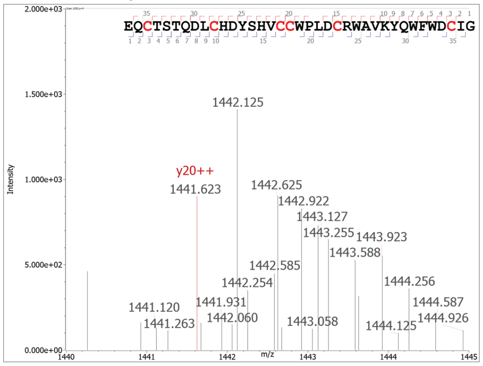

y21++ identifes C17-IAA

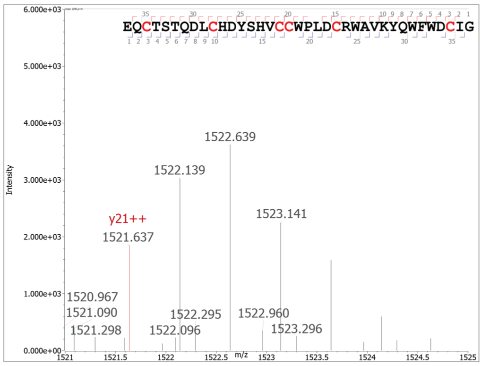

C Disulfide connectivity of GUR-1.6.12.2

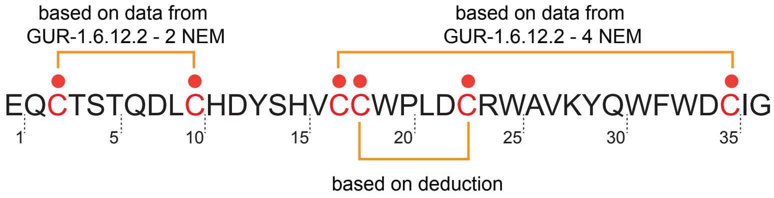

Figure S2. Identification of modified cysteines in selectively reduced GUR-1.6.12.2 samples by Electron-Transfer/Higher-Energy Collision Dissociation (EThcD) reveals disulfide connectivity. (A) MS2 spectra of the GUR-1.6.12.2 -2 NEM sample identifies NEM derivatized cysteines at positions C3 and C10, and IAA derivatized cysteines at the remaining positions. (B) MS2 spectra of the GUR-1.6.12.2 -4 NEM sample identifies IAA derivatized cysteines at positions C17 and C35, and NEM derivatized cysteines at the remaining positions. (C) Illustration of elucidated disulfide connectivity.

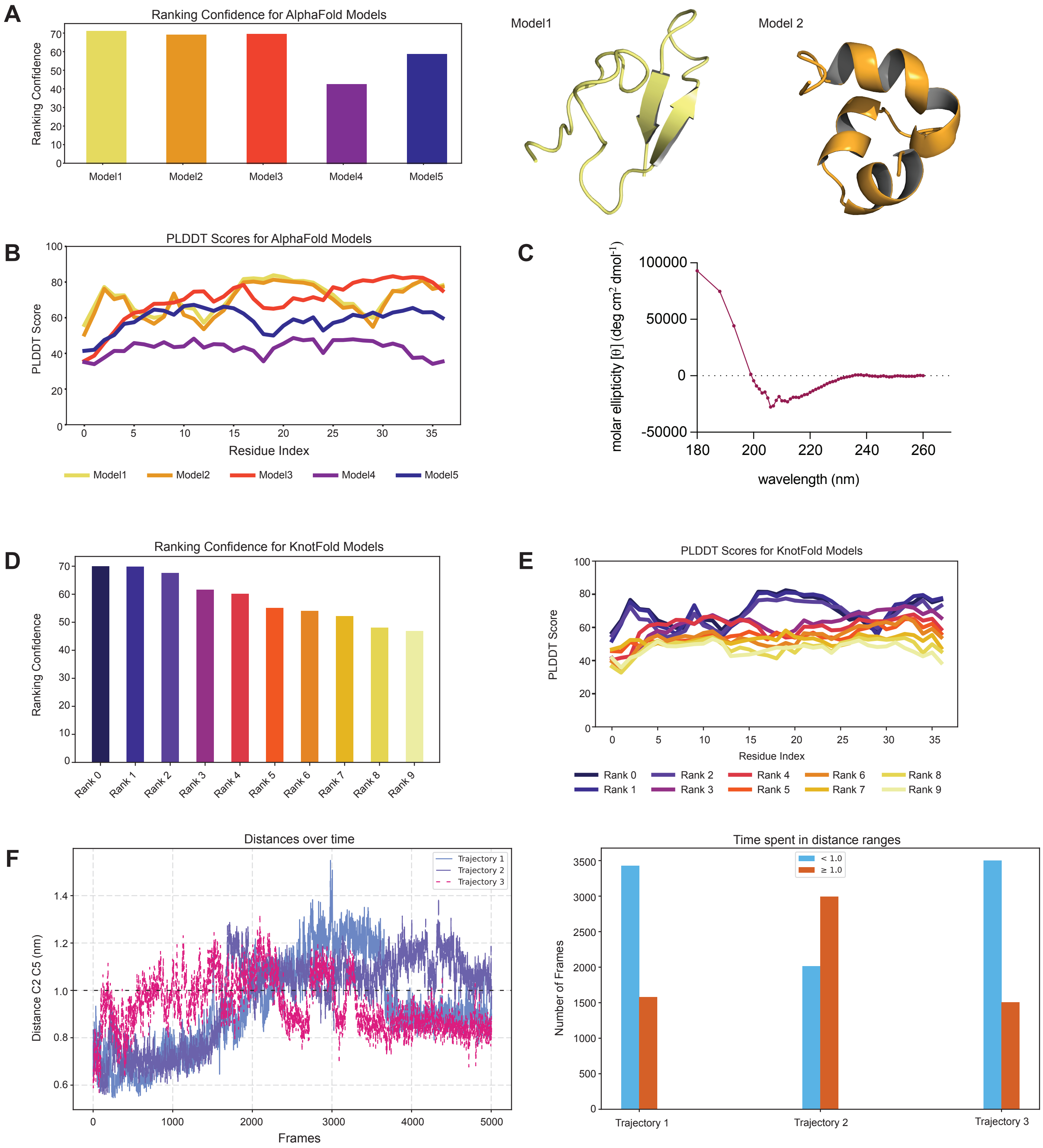
A
Ranking Confidence for AlphaFold Models
Ranking Confidence
Model1
Model2
Model3
Model4
Model5
Model1
Model 2
B
PLDDT Scores for AlphaFold Models
PLDDT Score
Residue Index
Model1
Model2
Model3
Model4
Model5
C
molar ellipticity [θ] (deg cm² dmol⁻¹)
wavelength (nm)
D
Ranking Confidence for KnotFold Models
Ranking Confidence
Rank 0
Rank 1
Rank 2
Rank 3
Rank 4
Rank 5
Rank 6
Rank 7
Rank 8
Rank 9
E
PLDDT Scores for KnotFold Models
PLDDT Score
Residue Index
Rank 0
Rank 1
Rank 2
Rank 3
Rank 4
Rank 5
Rank 6
Rank 7
Rank 8
Rank 9
F
Distances over time
Trajectory 1
Trajectory 2
Trajectory 3
Distance C2 C5 (nm)
Frames
Time spent in distance ranges
< 1.0
≥ 1.0
Number of Frames
Trajectory 1
Trajectory 2
Trajectory 3

Figure S3. Modeling of GUR-1.6.12.2 and prediction of its binding to RNF43. (A) AlphaFold models and their ranking confidence for GUR-1.6.12.2. (B) pLDDT values of residues for the AlphaFold models. (C) CD spectroscopy demonstrating the beta-sheet signature peak at approximately 218 nm. (D) Ranking confidence of KnotFold models using AlphaFold Model 1 and Model 2. (E) pLDDT scores of residues for the KnotFold models. (F) Distance between Cys II and Cys V over time across three different 50 ns trajectories, including the time spent in a closed conformation (distance < 1 nm, shown in blue) versus an open conformation (distance > 1 nm, shown in red) within individual trajectories.

SPR sensorgrams showing the binding of GUR-1.6.12.2 mutant peptides to RNF43

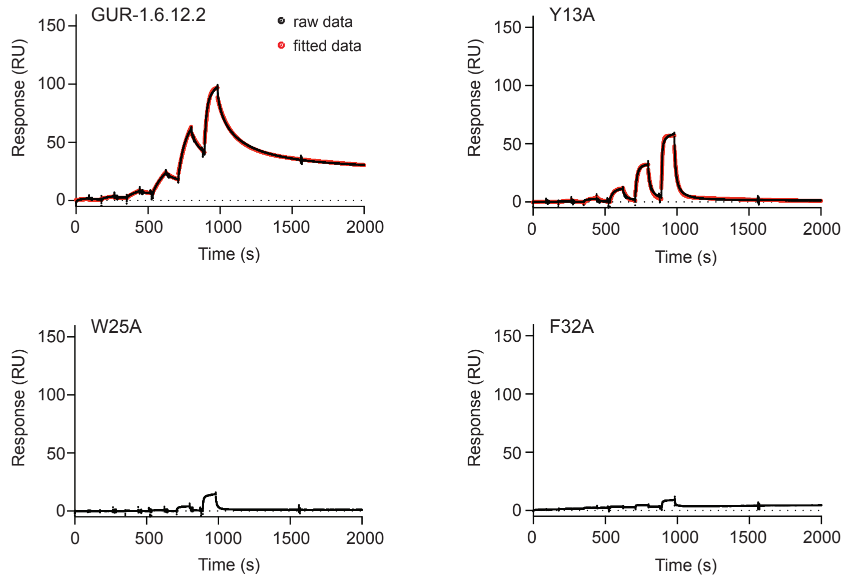

SPR sensorgrams showing the binding of GUR-1.6.12.2 truncated peptides to RNF43

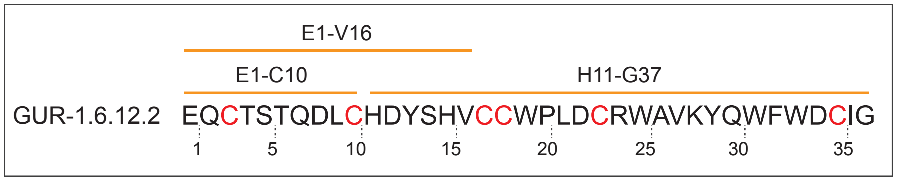

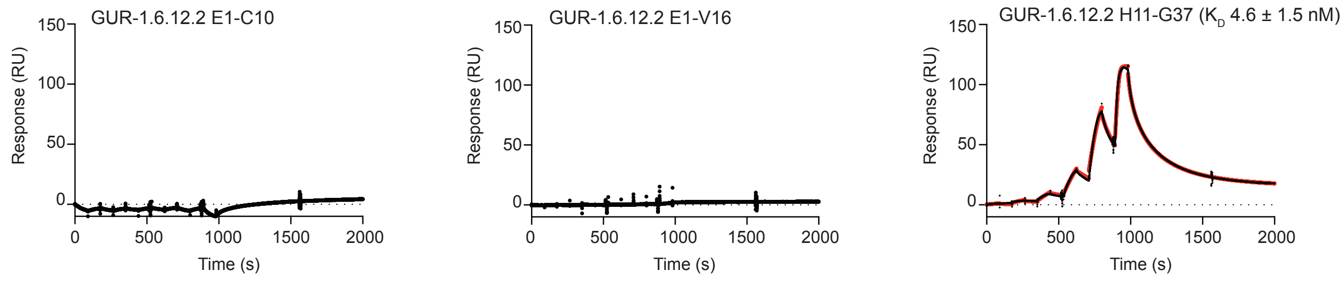

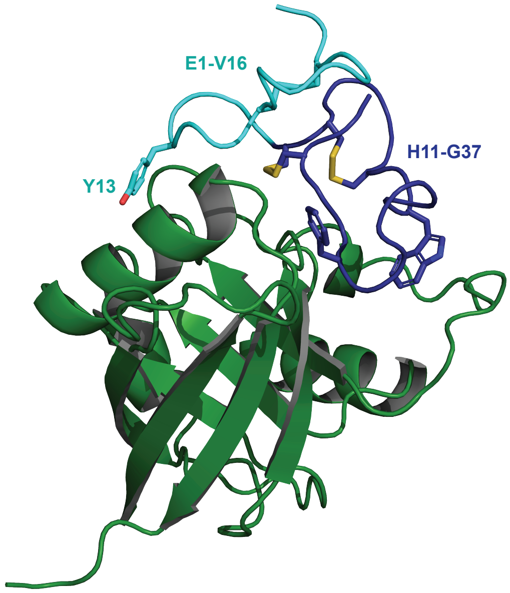

Figure S4. Representative SPR sensorgrams of GUR-1.6.12.2 mutant peptides. When the key residues were mutated to alanine, the binding affinity exhibited a significant reduction, validating the complex model (top four sensorgrams). The H11-G37 fragment of GUR-1.6.12.2 was found to be a main driving force for binding to RNF43 (bottom two sensorgrams), likely due to the segment C17-G37**.** SPR was independently performed at least three times (black: raw data; red: fitted data).

A

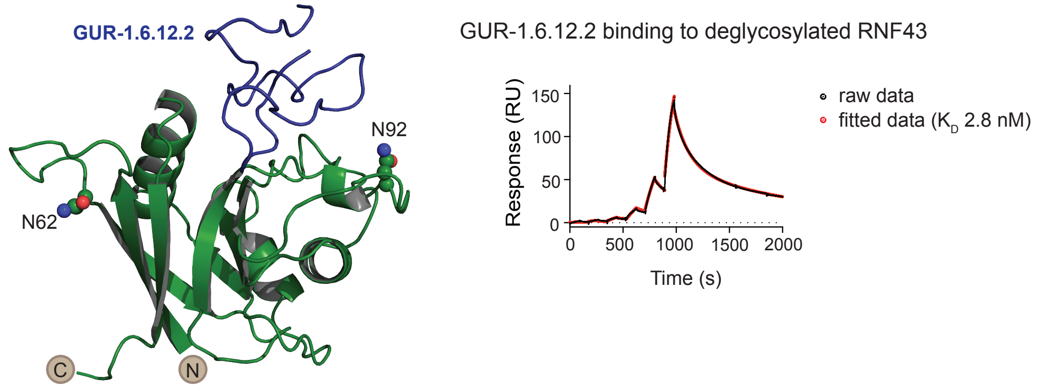

B

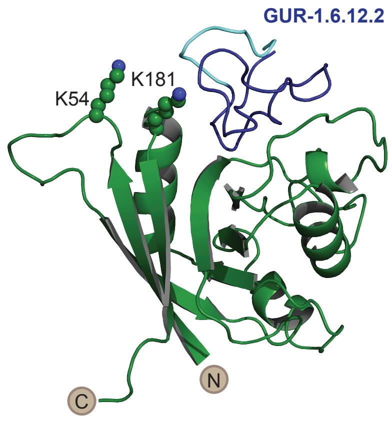

MeroX Cross-linking Table

PepSeq1	PepSeq2	LinkPos1	LinkPos2	Protein1	Protein2	Obs.m/z	Calc. m/z	Score
YKNQKA	{EQBT	K181	N-term	RNF43	DCP	713.338	1425.668	142
EPPAWPDY	{EQBTSTQD	Y197	N-term	RNF43	DCP	775.295	2251.897	197
{EQBTSTQD	KDPTG	N-term	K54	DCP	RNF43	885.359	1769.72	77

Figure S5. SPR data with deglycosylated RNF43 and cross-linking experiment support the complex model of GUR-1.6.12.2 with RNF43. (A) Representative SPR sensorgram showing the binding of GUR-1.6.12.2 to deglycosylated RNF43, with affinity remaining unchanged. Two glycosylated sites are presented in the complex model. SPR was independently performed at least three times (black: raw data; red: fitted data). (B) Potential cross-linking sites determined by mass spectrometry are summarized in the table. Scores greater than 100 equates to a false-positive rate of less than 2%, while scores ranging from 60 to 99 approximately equate to false-positive rate of 2-33%. Two lysine residues (K54 and K181) binding to the DCP are presented in the complex model. Y197 is not available in the crystal structure of RNF43 (PDB ID: 4KNG).

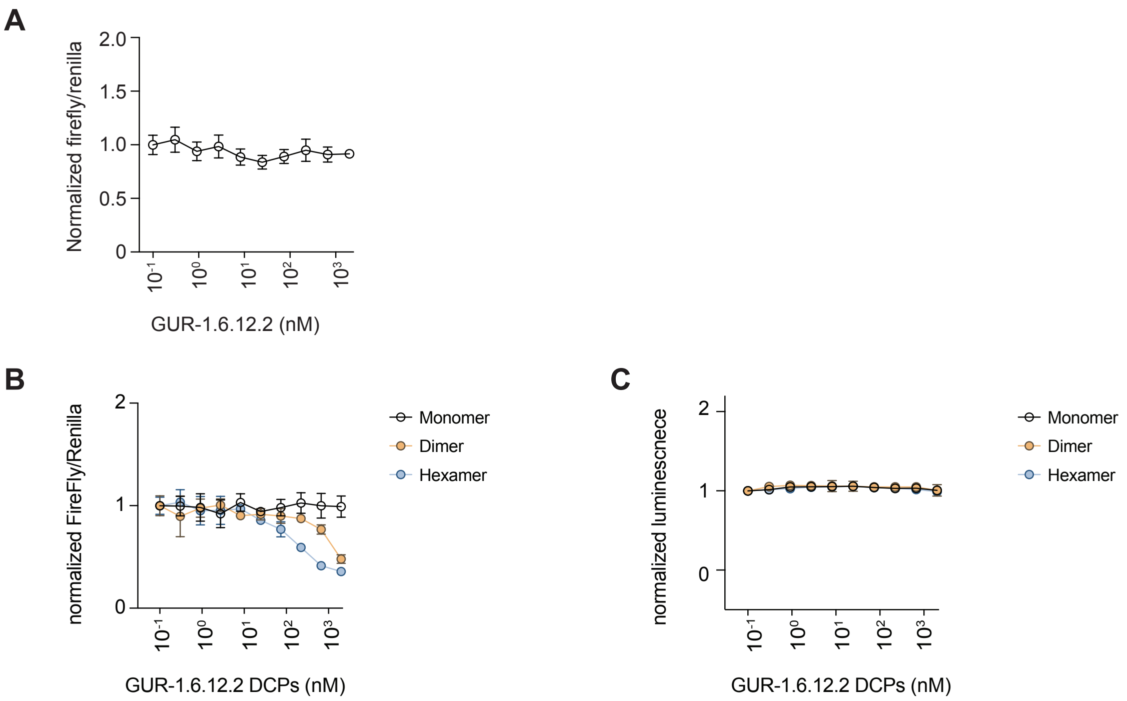

Figure S6. GUR-1.6.12.2 demonstrated minimal activity in the Wnt report assay. (A) The activity of GUR-1.6.12.2 was tested in the Wnt reporter assay (HEK293 TOPbrite luciferase reporter cells). Data were normalized to negative controls. Presented are mean ± SD (n = 3 per group) from a representative experiment. (B) Multivalent GUR-1.6.12.2 inhibited Wnt signaling activity without added RSPO2. (C) Multivalent GUR-1.6.12.2 did not cause cell death.
