# Supplementary material for: Selective and Potent Peptide Binders of RNF43 for Wnt Signaling Inhibition
Source: ACS Cent Sci. 2025 Jul 29;11(9):1670–81. doi: 10.1021/acscentsci.5c00744 (PMC12464775; doi:10.1021/acscentsci.5c00744)
Supplement: Supplementary file 2 [file oc5c00744_si_002.pdf]

Source Data

Selective and Potent Peptide Binders of RNF43 for Wnt Signaling Inhibition

Sunhee Hwang[1], Paula Flórez Salcedo[1], Antonion Korcari[2], John M. Nicoludis[3], Estefania Martinez Valdivia[1], Lingling Peng[1], Aaron T. Balana[1], Justin Mak[4], Christopher M. Crittenden[4], Amin Famili[4], Peter Liu[5], David Castillo-Azofeifa[2], Rami N. Hannoush[1], Stephen E. Miller[1], Christina I. Schroeder[1], and Xinxin Gao[1*]

Departments of [1]Peptide Therapeutics, [2]Regenerative Medicine, [3]Structural Biology, [4]Small Molecule Analytical Chemistry and Quality Control, [5]Microchemistry, Proteomics and Lipidomics, Genentech Inc., South San Francisco, CA, USA

*correspondence: gao.xinxin@gene.com

Address: 1 DNA Way, South San Francisco, California, USA

Keywords: disulfide-constrained peptide, Wnt signaling, E3 ubiquitin ligase, RNF43

Table of contents

Source Data-1. LC-MS analysis of peptides generated in Figure 1 and Figure 3.

Source Data-2. Representative chromatograms of peptide folding reaction.

Source Data-3. Original Western blot images for data in Figure 4D, and gel image of RNF43 deglycosylation experiments in Figure S5A.

Source Data-4. LC-MS analysis of the truncated peptides in Figure S4 and GUR-1.6.12.2 multimers.

Source data-1

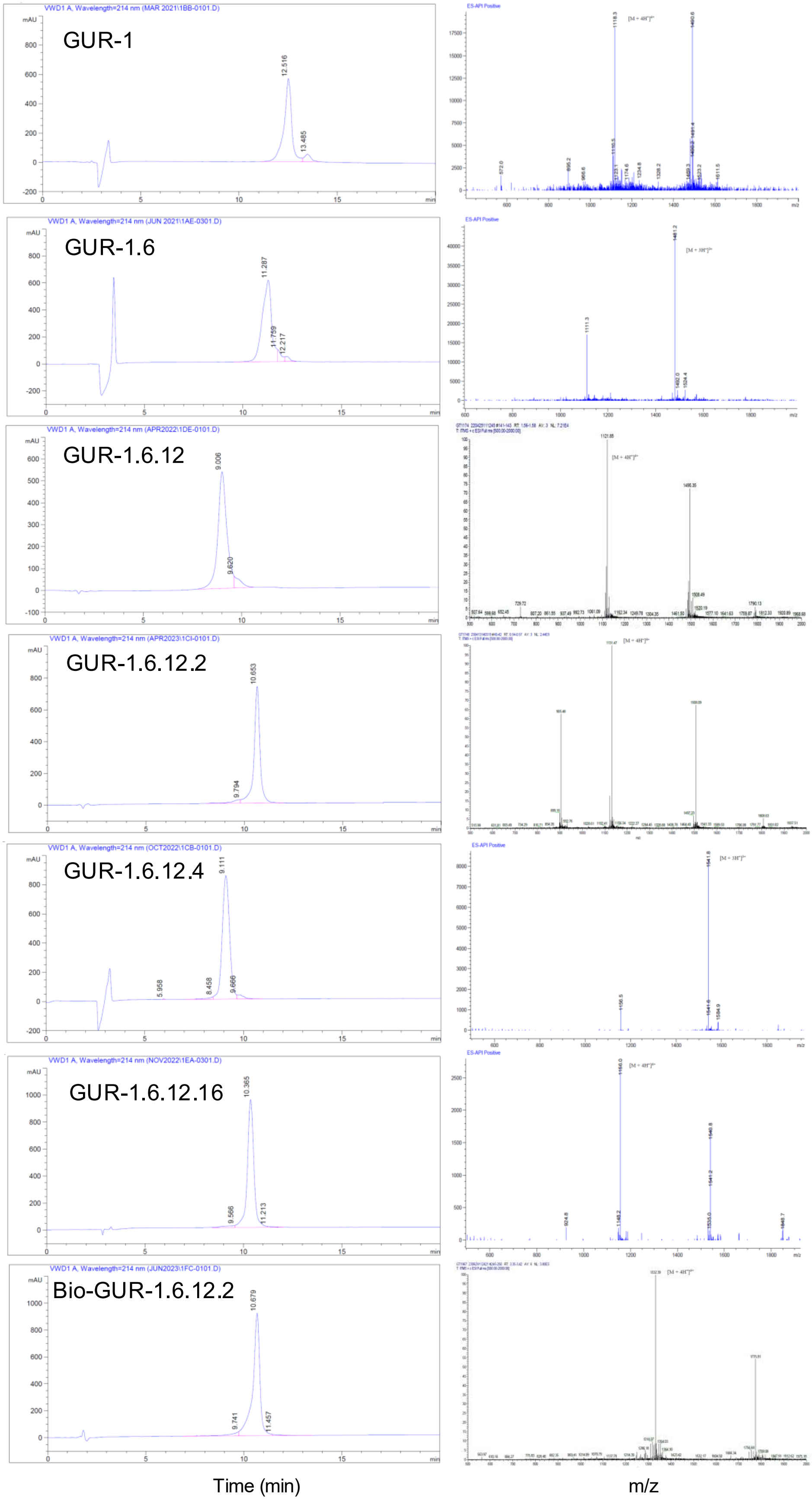

LC-MS analysis for all the peptides generated in the manuscript.

GUR-1.6.12.2 with F32A mutation

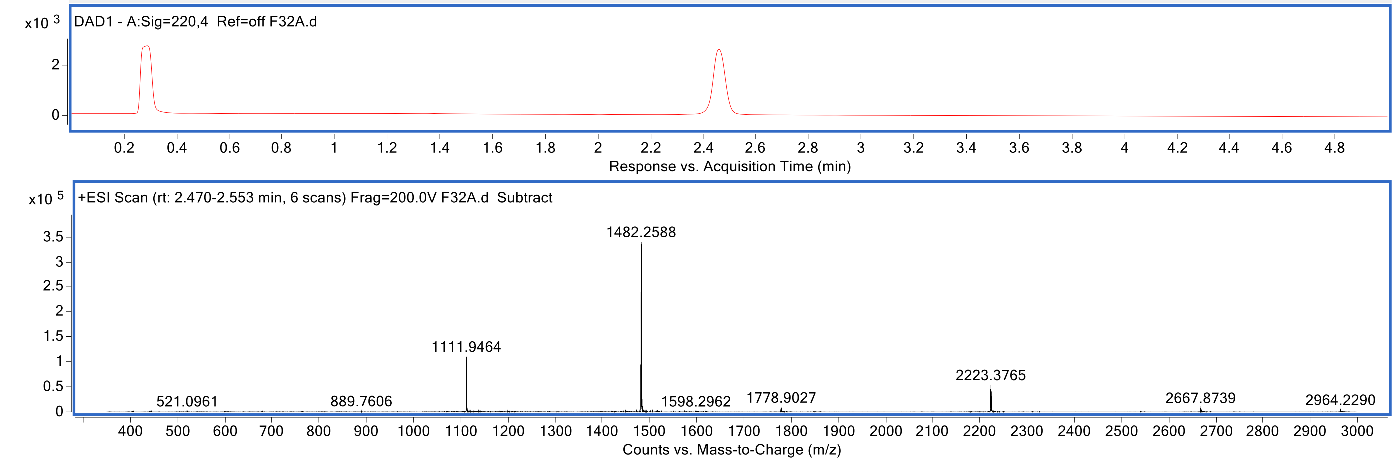

GUR-1.6.12.2 with Y13A mutation

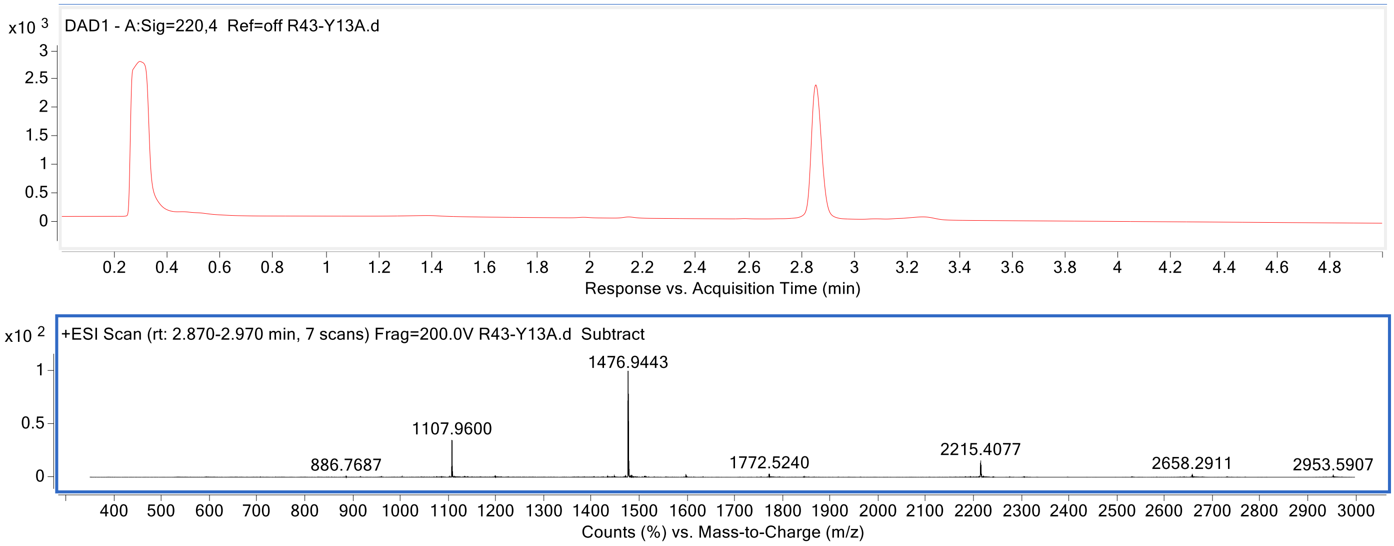

GUR-1.6.12.2 with W25A mutation

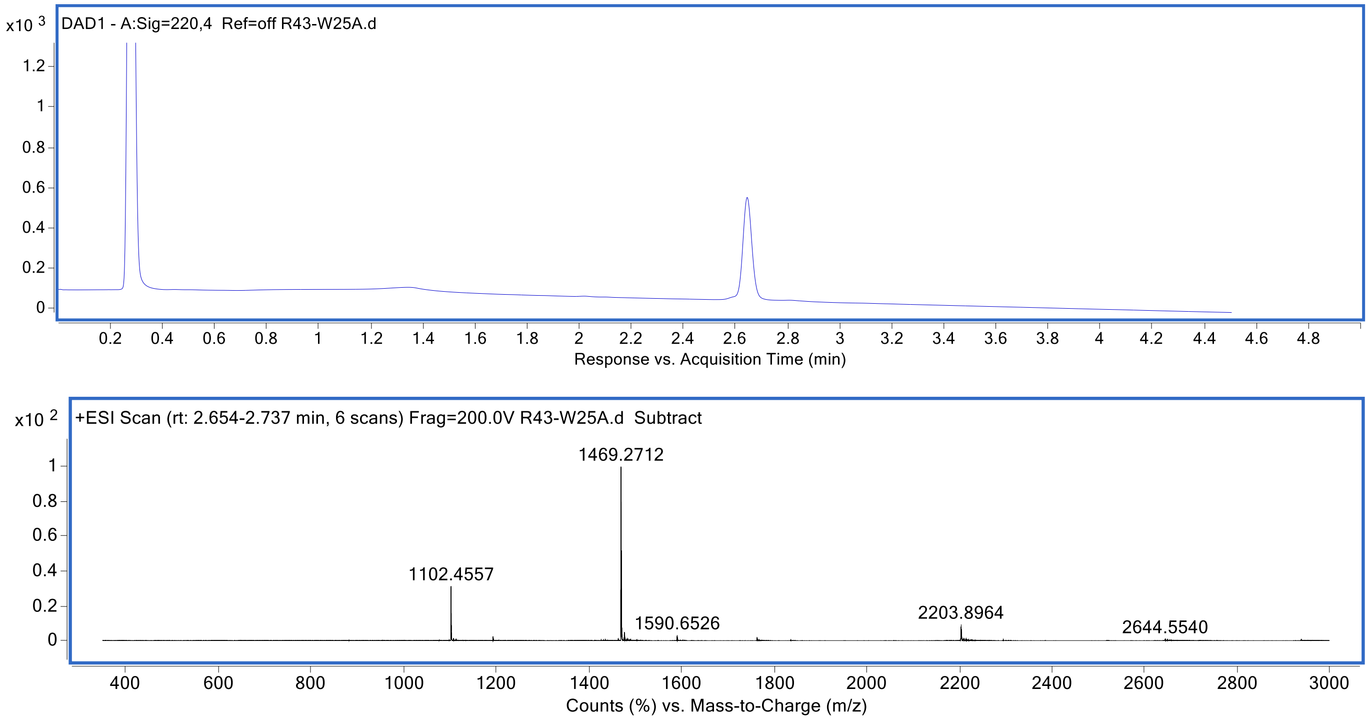

Source data-2

R43-GUR-1.6.12.4

Crude linear peptide HPLC analysis

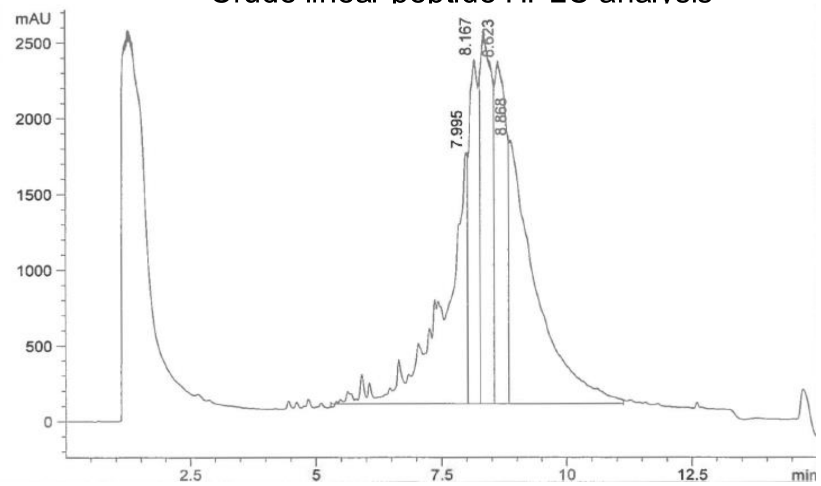

Folded DCP HPLC analysis (370 mg)

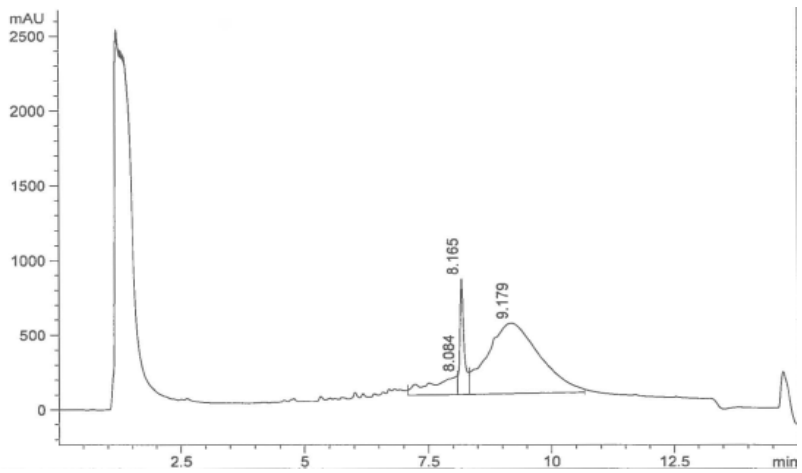

Folded and purified DCP HPLC and MS analysis (8 mg, total yield 2.1%)

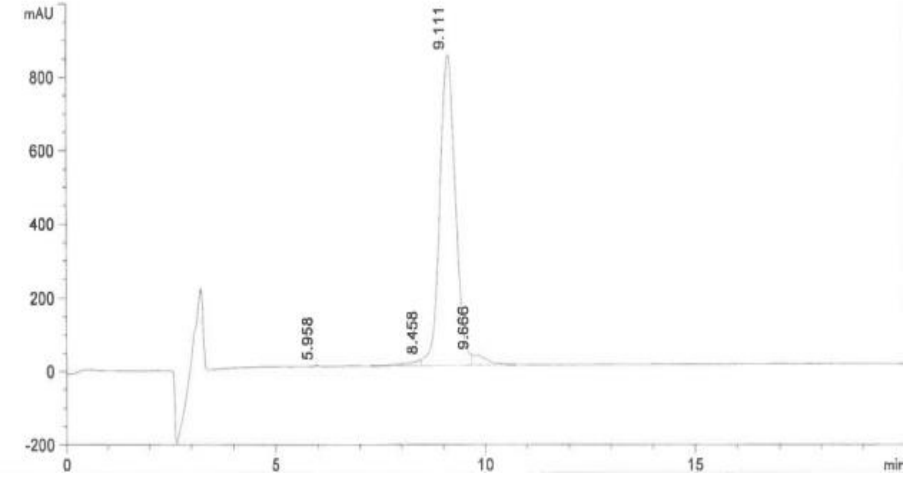

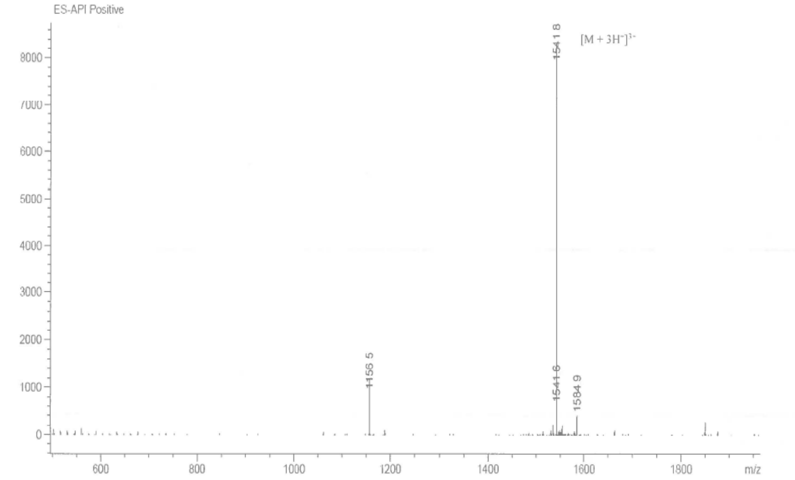

R43-GUR-1.6.12.16

Crude linear peptide HPLC analysis

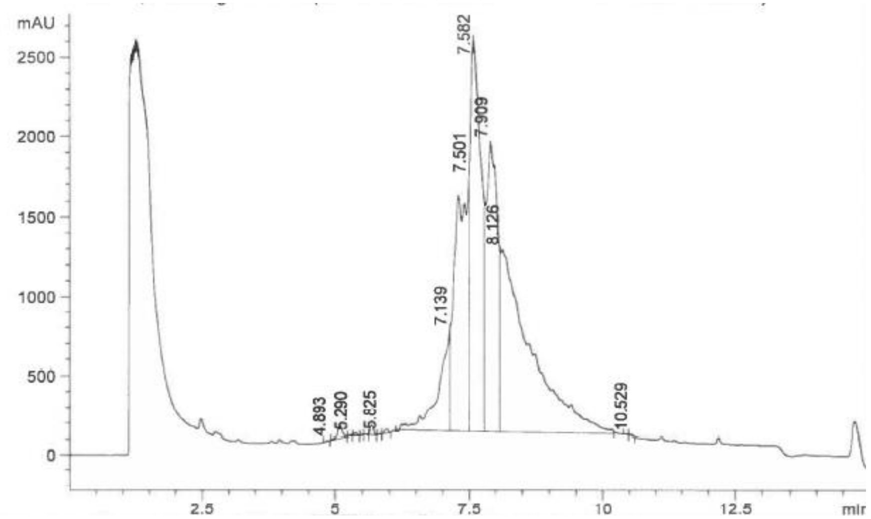

Folded DCP HPLC analysis (370 mg)

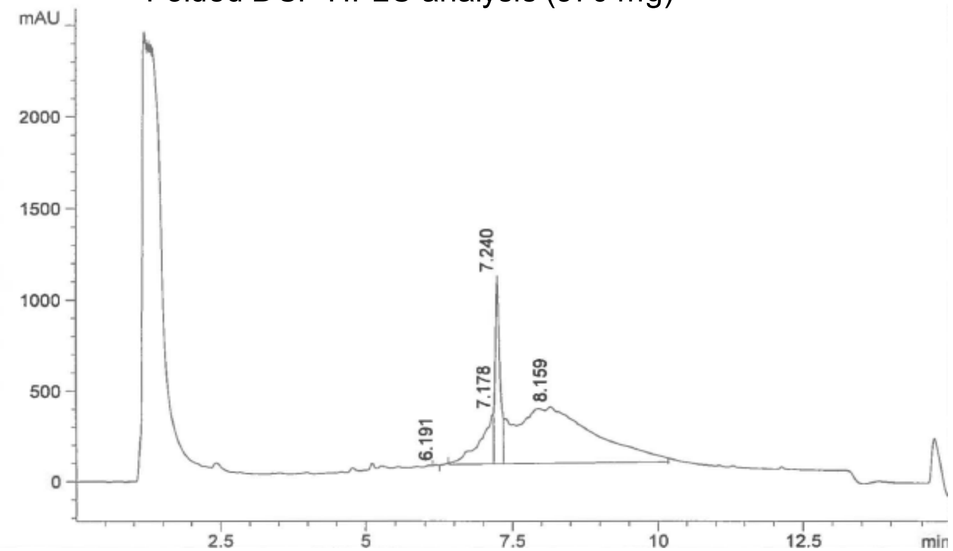

Folded and purified DCP HPLC and MS analysis (6 mg, total yield 1.6%)

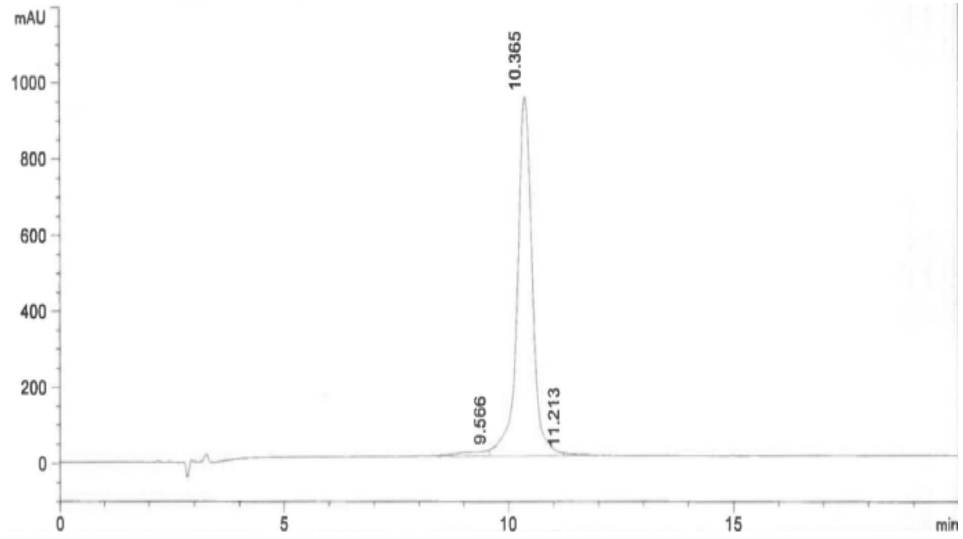

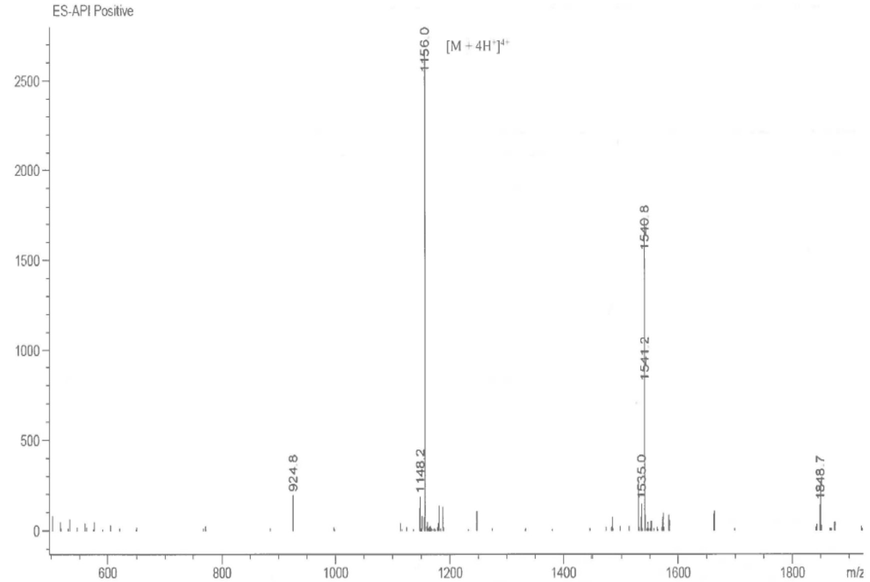

Source Data-3

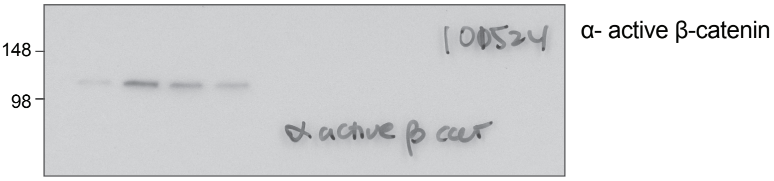

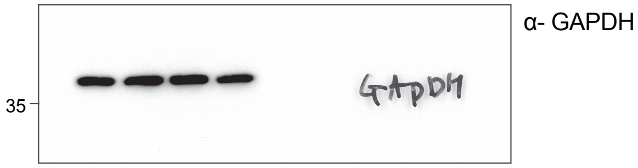

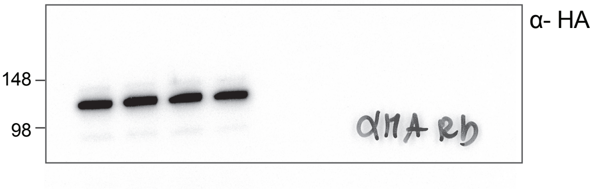

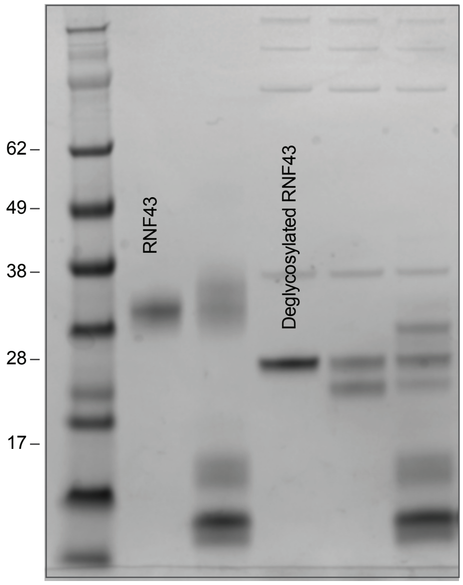

Coomassie staining

Source data-4

GUR-1.6.12.2 E1-C10 fragment peptide

Analytical LC:

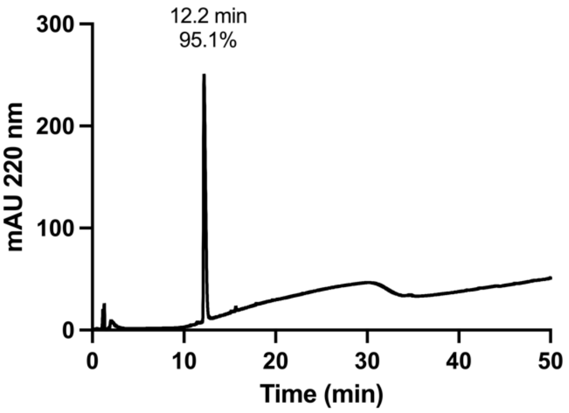

Deconvoluted mass:

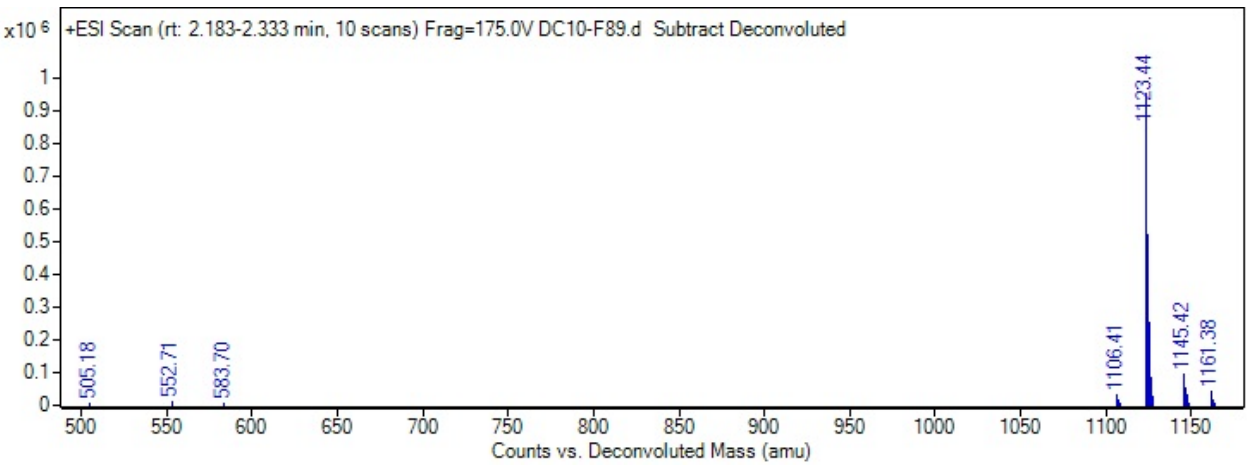

GUR-1.6.12.2 E1-V16 fragment peptide

Analytical LC:

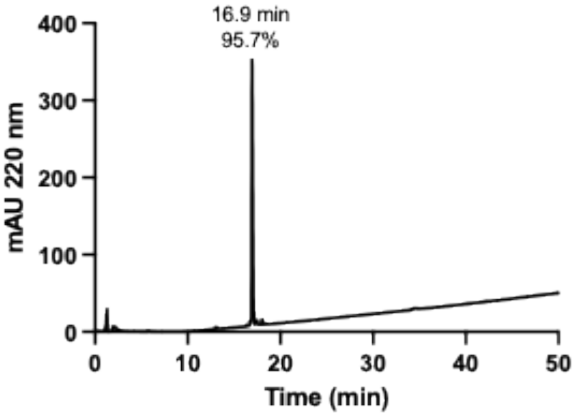

Deconvoluted mass:

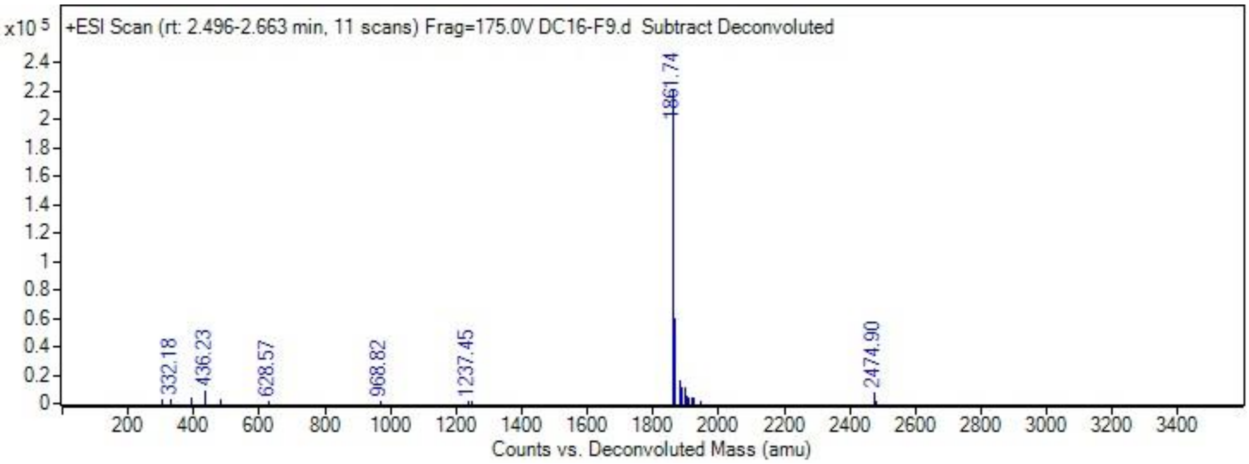

GUR-1.6.12.2 H11-G37 fragment peptide

Analytical LC:

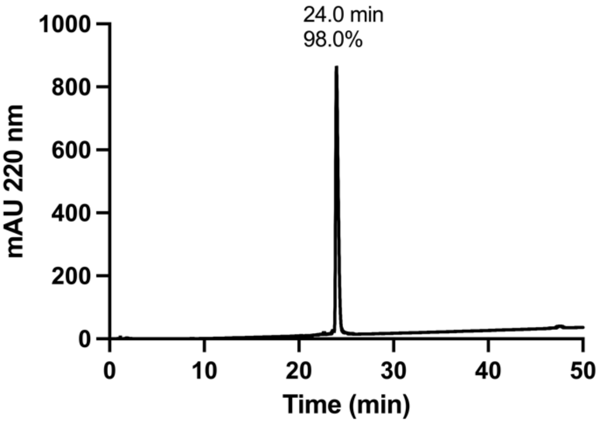

Deconvoluted mass:

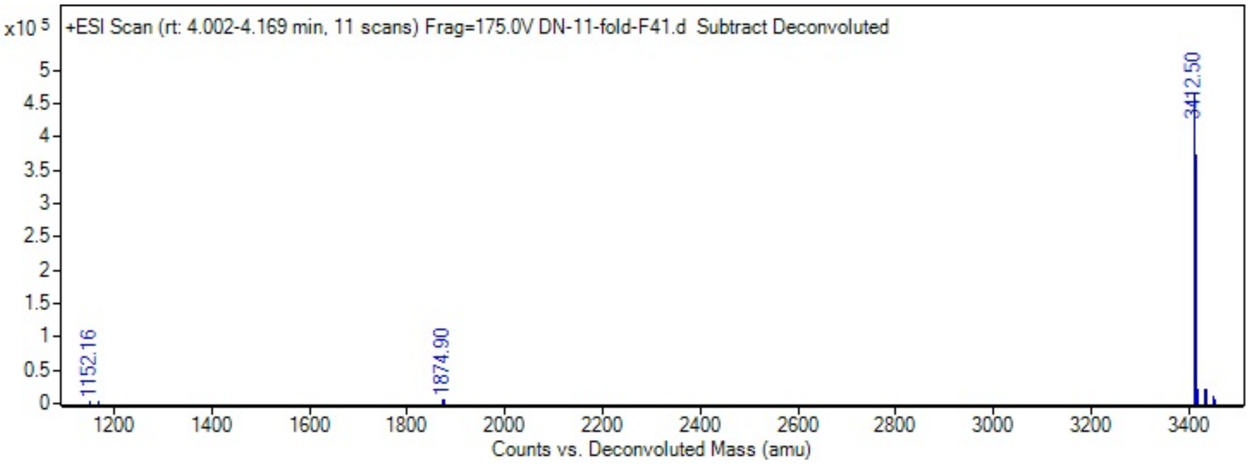

di-GUR-1.6.12.2

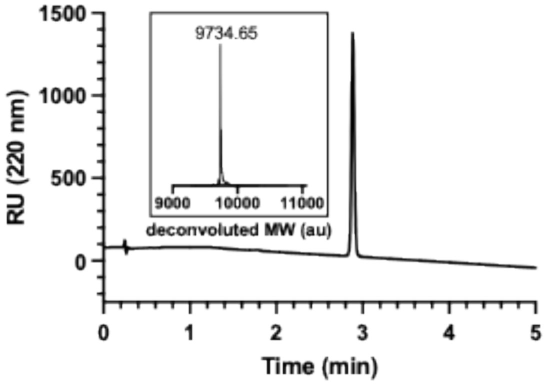

hex-GUR-1.6.12.2

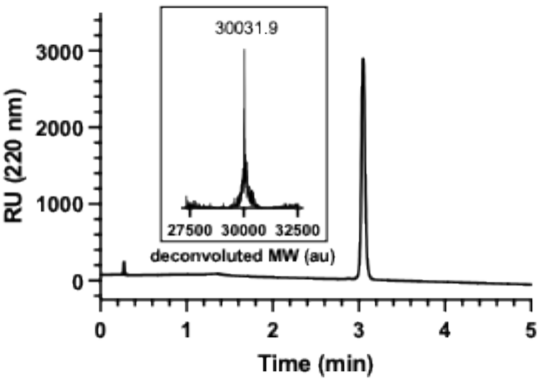

Analytical LC with deconvoluted mass information
